# Efficacy and Safety of Lisdexamfetamine Versus Topiramate Versus Naltrexone/Bupropion in Individuals With Binge Eating Disorder: A Network Meta‐Analysis

**DOI:** 10.1002/erv.70035

**Published:** 2025-09-22

**Authors:** Tamer Hodrob, Ibrahim Ismail, Alaaeddin Abusalameh, Celina R. Andonie, Omar Ayesh, Hazem Ayesh

**Affiliations:** ^1^ Faculty of Medicine Al‐Quds University East Jerusalem Palestine; ^2^ Department of Medical Laboratory Sciences Al‐Aqsa University Gaza Palestine; ^3^ Deaconess Health System Evansville Indiana USA

**Keywords:** binge eating disorder, lisdexamfetamine, naltrexone/bupropion, topiramate, weight

## Abstract

**Objective:**

To compare the efficacy and safety of lisdexamfetamine, topiramate, and naltrexone/bupropion for treating binge eating disorder (BED) using network meta‐analysis.

**Method:**

We systematically searched PubMed, Scopus, ClinicalTrials.gov, and Cochrane Central up to February 2025 for randomized controlled trials evaluating these medications versus placebo in adults with BED. Primary outcomes were changes in binge eating frequency and body weight; secondary outcomes included serious adverse events, discontinuation rates, and common side effects. A frequentist random‐effects network meta‐analysis was performed.

**Results:**

Twelve trials (*n* = 1988) met inclusion criteria. Both lisdexamfetamine (MD −1.61, 95% CI: −2.41 to −0.81) and topiramate (MD −1.63, 95% CI: −2.53 to −0.74) significantly reduced binge eating frequency versus placebo, with comparable efficacy. Topiramate produced the greatest weight loss (MD −5.5 kg), followed by lisdexamfetamine (−4.6 kg). Naltrexone/bupropion did not significantly reduce binge frequency (MD −2.07, 95% CI: −4.45 to 0.31). Lisdexamfetamine was associated with higher risks of dry mouth and gastrointestinal events. No significant increase in serious adverse events was observed for any medication.

**Conclusions:**

Topiramate and lisdexamfetamine are effective for reducing binge episodes and weight in BED. Naltrexone/bupropion showed modest weight effects but lacked clear efficacy for binge reduction. These findings support topiramate and lisdexamfetamine as primary pharmacologic options for BED.

## Highlights

- Both lisdexamfetamine and topiramate significantly reduce binge eating frequency and promote weight loss in adults with binge eating disorder.
- Naltrexone/bupropion does not show significant efficacy for reducing binge eating episodes, though it is associated with modest weight loss.
- Lisdexamfetamine is associated with increased dry mouth and gastrointestinal side effects; serious adverse events were not significantly increased for any treatment.

## 1 Introduction

Binge‐eating disorder (BED) is a prevalent psychiatric disorder, affecting approximately 1.4% of the population worldwide (Kessler et al. 2013). Characterised by recurrent lack of control and consuming larger amounts of food beyond what most people would eat in similar situations (Table 1, DSM‐IV and DSM‐5 Diagnostic Criteria for binge‐eating Disorder 2015). BED is associated with significant physical and psychological consequences, including obesity, depression, and an increased risk of metabolic syndrome, all of which place a burden on healthcare systems (Udo and Grilo 2019; Hudson et al. 2010; Streatfeild et al. 2021). In addition to these metabolic and psychiatric comorbidities, BED has been linked to distorted body image, elevated risk of suicidal ideation, and higher rates of alexithymia, further intensifying its psychological burden and clinical complexity (Carano et al. 2006, 2012). Despite its growing recognition as a public health concern, pharmacological treatment options remain limited. Currently, lisdexamfetamine, a central nervous system stimulant is the only FDA‐approved medication for BED (Heo and Duggan 2017), while off‐label treatments such as topiramate, an anticonvulsant and a combination of naltrexone and bupropion, a combination of an opioid receptor antagonist and an atypical antidepressant, have shown promise in clinical trials (McElroy et al. 2003; Grilo et al. 2023).

**TABLE 1:** Study characteristics and outcomes in the included clinical trials the table summarises various BED clinical trials, detailing study design, registration, duration, treatment arms, primary outcomes, and population characteristics.

Study	Design	Registration	Duration	Treatment arms	Primary outcomes	Population
McElroy, Hudson, Mitchell, et al. (2015)	Phase 2, RCT, DB, PC	NCT01291173	11 weeks	Lisdexamfetamine (30 mg, 50 mg, 70 mg daily), placebo	Change in number of BE days/week	BED, aged 18–55 years, BMI (25–45)
McElroy, Hudson, Ferreira‐Cornwell, et al. (2015)	Phase 3, RCT, DB, PC	NCT01718483	11–12 weeks	Lisdexamfetamine (30–70 mg daily), placebo	Change in number of BE days/week	BED, aged 18–55 years, ≥ 3 BE days/week for 2 weeks, BMI (18–45)
McElroy, Hudson, Ferreira‐Cornwell, et al. (2015)	Phase 3, RCT, DB, PC	NCT01718509	11–12 weeks	Lisdexamfetamine (30–70 mg daily), placebo	Change in number of BE days/week	BED, aged 18–55 years, ≥ 3 BE days/week for 2 weeks, BMI (18–45)
Guerdjikova et al. (2016)	Phase 3, RCT, DB, PC	NCT01090713	12 weeks	Lisdexamfetamine (30–70 mg daily), placebo	Change in number of BE days/week	BED, aged 18–55 years, ≥ 3 BE days/week for 2 weeks, BMI (18–45)
Grilo et al. (2024)	Phase 3, RCT, DB, PC	NCT03926052	12 weeks	Lisdexamfetamine (30–70 mg daily), placebo	Change in EDE BE (BE frequency)	BED, aged 18–64 years, ≥ 65% reduction in binge‐eating after CBT and LDX (Stage 1 responders), BMI (27–50)
Grilo et al. (2021)	NR, pilot RCT, DB, PC	NCT02317744	26 weeks	Naltrexone/bupropion XL 50/300 mg daily, placebo	Change in BE frequency	BED, aged 18–65 years, BMI (30–50)
Grilo et al. (2022)	Phase 2 Phase 3, RCT, DB, PC	NCT03045341	16 weeks	Naltrexone/bupropion SR 32/360 mg daily, BWL, placebo	Change in EDE BE (BE frequency), weight	BED, aged 18–70 years, BMI (27–50)
Grilo, Lydecker, Jastreboff, et al. (2023)	Phase 2 Phase 3, RCT, DB, PC	NCT03539900	12 weeks	Naltrexone/bupropion SR 32/360 mg daily, placebo	Change in EDE BE (BE frequency), weight, BMI	BED, aged 18–70 years, BMI (27–50),
Grilo, Lydecker, Jastreboff, et al. (2023)	Phase 3, RCT, DB, PC	NCT03047005	16 weeks	Naltrexone/bupropion SR 32/360 mg daily, placebo	Change in BE frequency, weight	BED, aged 18–70 years, ≥ 65% reduction in binge‐eating after NB or NB and BWL (Stage 1 responders), BMI (21.5–50),
McElroy et al. (2003)	Phase 3, RCT, DB, PC	NR	14 weeks	Topiramate 25–600 mg daily, placebo	Change in number of BE episodes/week	BED, aged 18–60 years, Y‐BOCS‐BE score ≥ 15, BMI ≥ 30
McElroy et al. (2007)	Phase 2 Phase 3, RCT, DB, PC	NCT00210808	16 weeks	Topiramate 25–400 mg daily, placebo	Change in number of BE days/week	BED, aged 18–65 years, ≥ 3 BE days/week for 2 weeks, BMI (30–50)
Claudino et al. (2007)	Phase 3, RCT, DB, PC	NCT00307619	21 weeks	Topiramate 25–200 mg daily, CBT, placebo	Change in weight	BED, aged 18–60 years, BES score > 17, BMI ≥ 30

Abbreviations: BE, Binge Eating; BED, binge eating disorder; BES, Binge Eating Scale; BMI, Body Mass Index; BWL, Behavioural Weight Loss; CBT, Cognitive Behavioural Therapy; DB, double‐blind; EDE, Eating Disorder Examination; LDX, lisdexamfetamine; NB, Naltrexone/Bupropion; PC, placebo‐controlled; RCT, randomized controlled trial; Y‐BOCS‐BE, Yale‐Brown Obsessive Compulsive Scale modified for Binge Eating.

While non‐pharmacological treatments—such as cognitive‐behavioural therapy, interpersonal therapy, and behavioural weight loss therapy—are recommended as first‐line therapeutic options (Davis et al. 2020), several pharmacologic agents targeting various neurotransmitters —particularly lisdexamfetamine and topiramate—have shown promising efficacy in reducing binge frequency and associated psychopathology (Boswell et al. 2020). However, current evidence is limited by multiple conflicting findings, including heterogeneous outcome measures, absence of long‐term follow‐up, limited relapse prevention seen with lisdexamfetamine (Grilo et al. 2021), and high dropout rates due to adverse events with topiramate (McElroy et al. 2003, 2004), all of which complicate treatment decisions. Additionally, previous pairwise meta‐analyses often combined pharmacological and psychotherapeutic trials (Brownley et al. 2016; Peat et al. 2017), reducing the generalisability and conclusions of the most effective pharmacologic treatment for BED.

In this network meta‐analysis, we aim to evaluate the efficacy and safety of lisdexamfetamine in comparison with other pharmacologic agents for the treatment of BED. We focus on key outcomes such as reduction in binge eating episodes (frequency), change in weight (kg), and adverse events.

To date, Comprehensive network meta‐analyses directly comparing lisdexamfetamine, topiramate, and naltrexone/bupropion for the treatment of binge‐eating disorder (BED) remain scarce, creating uncertainty in clinical decision‐making regarding the most effective and safest pharmacologic options. A recent systematic review and network meta‐analysis by Costa et al. identified lisdexamfetamine and topiramate as the most effective agents for reducing binge‐eating frequency, while naltrexone/bupropion demonstrated limited efficacy (Costa et al. 2025). However, that analysis did not include three additional randomized controlled trials evaluating these medications and did not comprehensively assess safety outcomes, such as serious adverse events, alongside efficacy. To address these gaps, our network meta‐analysis incorporates all available data and simultaneously evaluates both efficacy and safety profiles across a broader evidence base. This analysis will aid clinicians in making informed decisions, and ultimately enhance the overall management of BED.

## 2 Methods

This systematic review and network meta‐analysis was conducted in accordance with the Preferred Reporting Items for Systematic Reviews and Meta‐Analyses (PRISMA) guidelines (Page et al. 2021). The PRISMA checklist was followed throughout the study (Supporting Information S1: Supplement S10). The protocol was prospectively registered on the Open Science Framework (OSF; Registration https://doi.org/10.17605/OSF.IO/STJBH) (Hodrob and Ayesh 2025).

### 2.1 Search Strategy

We systematically searched PubMed, Scopus, ClinicalTrials.gov, and the Cochrane Central Register of Controlled Trials to identify relevant studies from inception to February 08, 2025. The search terms included: ((‘Binge Eating Disorder’ OR BED OR ‘Eating Disorder’) AND (‘Lisdexamfetamine’ OR ‘Vyvanse’ OR ‘Topiramate’ OR ‘Topamax’ OR ‘Trokendi XR’ OR ‘Qudexy XR’ OR ‘Naltrexone’ OR ‘ReVia’ OR ‘Vivitrol’ OR ‘Depade’ OR ‘Bupropion’ OR ‘Wellbutrin’ OR ‘Wellbutrin SR’ OR ‘Wellbutrin XL’ OR ‘Aplenzin’ OR ‘Forfivo XL’ OR ‘Zyban’ OR ‘Contrave’) AND (‘randomized controlled trial’ OR RCT OR ‘clinical trial’ OR ‘trial’). The detailed search strategy is reported in Supporting Information S1: Supplement 1. No language or publication status restrictions were applied.

### 2.2 Screening Process

Two reviewers (TH and II) independently screened titles and abstracts, followed by full‐text reviews to identify eligible studies. Discrepancies were resolved by consensus or by a third reviewer if necessary.

### 2.3 Study Selection

We included randomized controlled trials (RCTs) assessing pharmacologic therapies in adult patients with binge eating disorder (BED). Eligible interventions included Lisdexamfetamine, topiramate and naltrexone/bupropion, that were used for treatment of BED. Comparators included placebo. Studies were required to report on at least binge eating episodes (frequency). Reporting on weight change and adverse events was preferred but not mandatory for inclusion; studies missing one or more secondary outcomes were not excluded. Trials focusing exclusively on paediatric populations or non‐pharmacologic interventions, or did not report relevant outcomes, or were non‐comparative studies or conference abstracts without full‐text availability were excluded.

### 2.4 Data Extraction

Two independent reviewers extracted data using a standardized and piloted form. Collected variables included participant demographics, baseline characteristics, intervention details, and outcome measures. Specifically, data on age, sex, weight (kg), body mass index (BMI), and binge eating episodes/week were collected. Information on study design, duration, and adverse events were also extracted (Supporting Information S1: Supplement S3). Combined means and standard deviations were calculated following the Cochrane Handbook for Systematic Reviews of Interventions guidelines (Cochrane Handbook for Systematic Reviews of Interventions).

To ensure consistency across studies, all binge eating episode data were standardized to episodes per week. For studies using assessment tools such as the Eating Disorder Examination (EDE) with longer recall periods, reported frequencies were converted to weekly averages. Data reported as binge episodes per week were used directly. When food diaries or other supplementary confirmation methods were used, those data were incorporated as originally reported.

The Cochrane Risk of Bias (ROB) tool was used to assess study quality, with each domain scored as 1 (low), 2 (moderate), or 3 (high) risk. We also evaluated overall confidence in network estimates using the CINeMA (Confidence in Network Meta‐Analysis) framework (Nikolakopoulou et al. 2020).

### 2.5 Statistical Analysis

We performed a network meta‐analysis to evaluate several therapies by analysing data from multiple trials, allowing for both direct and indirect comparisons. This method synthesises data to determine the relative effectiveness of each therapy, even though some were not directly compared in any one trial (Cochrane Handbook for Systematic Reviews of Interventions). To account for study heterogeneity, a random‐effects model was employed in the statistical analysis. Mean differences (MD) for continuous outcomes and relative risks (RR) for dichotomous outcomes were calculated along with 95% CIs. The reference comparator was placebo. The highest tolerated dose was used for primary analysis when there were multiple doses available for an intervention. For outcomes with various measurement techniques, we used the standardized mean difference (SMD). Heterogeneity was assessed using tau‐squared (τ^2^), which measures between‐study variance; I‐squared (I^2^), which estimates the proportion of total variability due to heterogeneity; and Q statistics, which test for the presence of heterogeneity. In regard to thresholds, we interpreted I^2^ values as follows: 0%–25% indicated minimal heterogeneity, 25%–50% moderate heterogeneity, and above 50% high heterogeneity (Higgins et al. 2003). We pre‐specified to examine heterogeneity by key study characteristics, including age, sex, BMI, and study duration. The meta and netmeta packages in RStudio were used for all analyses (Download RStudio; Balduzzi et al. 2023). Treatments were ranked using P‐scores, which estimate the probability that an intervention is among the most effective (Rücker and Schwarzer 2015). Transitivity—the assumption that studies are sufficiently similar in effect modifiers—was assessed by comparing baseline covariates (age, BMI, sex) across treatment comparisons using ANOVA and boxplots visualizations. Global inconsistency was evaluated via design‐by‐treatment interaction model.

The outcomes evaluated include the following: binge eating episodes (frequency), change in weight (kg). Safety outcomes include serious adverse events, treatment discontinuation due to TEAEs (treatment‐emergent adverse events), and the frequency of headache, dry mouth, and gastrointestinal (GI) adverse events including nausea, vomiting, GI disturbances, decreased appetite, dyspepsia, diarrhoea, and/or constipation among participants. Sensitivity analyses included exclusion of high‐risk bias studies and leave‐one‐out analyses to evaluate the influence of individual trials on overall estimates (Supporting Information S1: Supplement S9). Publication bias was assessed using funnel plots and Egger's test for the primary outcomes.

### 2.6 Bias Assessment and Certainty of Evidence

The risk of bias was assessed using the RoB 2 (Risk of Bias 2) tool, which evaluates bias across six domains: randomisation, allocation concealment, blinding of participants and/or outcome investigators, missing outcome data, outcome measurement, and selection of reported results (Sterne et al. 2019). Two authors (TH and II) independently assessed the risk of bias. Disagreements were resolved through discussion or by consulting a third author (AA). The certainty of evidence was assessed using the Confidence in Network Meta‐Analysis (CINeMA) framework (Supporting Information S1: Supplement S8) (Nikolakopoulou et al. 2020).

## 3 Results

### 3.1 Study Characteristics

**FIGURE 1:**
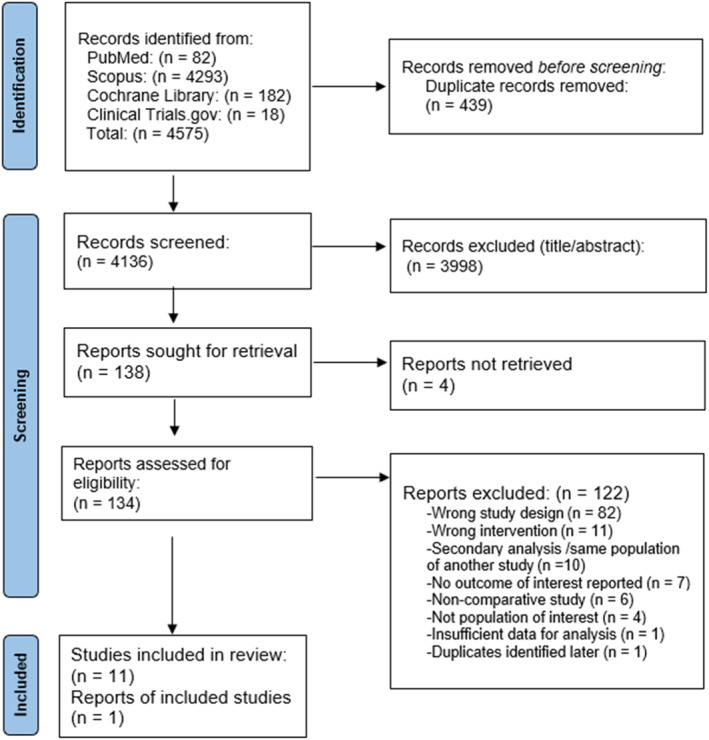
PRISMA flowchart for study selection.

**FIGURE 2:**
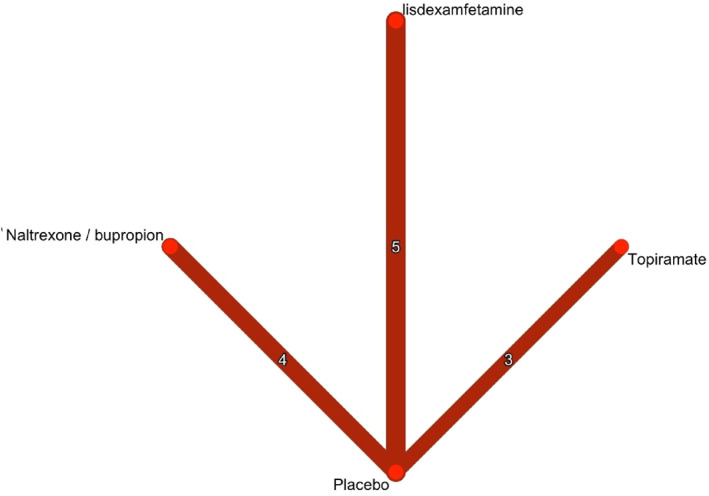
Network plot of treatment comparisons for binge episodes (frequency). This network plot shows the direct comparisons among, lisdexamfetamine, naltrexone/bupropion, and topiramate and placebo in studies for binge eating disorder. The numbers in the edges reflect the number of studies involving each treatment.

A total of 4575 records were identified through database searches. of which 134 full‐text articles were assessed for eligibility. Ultimately, 12 randomized controlled trials (RCTs) (McElroy et al. 2003, 2015; McElroy, Hudson, Ferreira‐Cornwell, et al. 2015; Guerdjikova et al. 2016; Grilo et al. 2024, 2021, 2022, 2023; McElroy et al. 2007; Claudino et al. 2007), and one additional report met the inclusion criteria (McElroy et al. 2017) (Figure 1), comprising data from a total of 1988 patients. These studies investigated the efficacy of lisdexamfetamine, topiramate, naltrexone/bupropion, and placebo (Figure 2). The duration of the trials ranged from 11 to 26 weeks. All participants met the diagnostic criteria for binge eating disorder (BED) as defined by either the DSM‐IV or DSM‐5 (Table 1). The mean age of participants was 42.8 years (SD 11.67), with a mean weight of 102.38 kg (SD 21.2). The mean number of baseline binge episodes was 7.58 per week (SD 5.72); baseline characteristics are summarised in Table 2. The risk of bias was generally rated as some concerns, with some domains having low risk (Supporting Information S1: Supplement S4). Publication bias was observed for the primary outcomes (binge episodes and weight), as well as for some safety outcomes (dry mouth and GI adverse events). While other safety outcomes showed a low risk of bias. The certainty of evidence was generally low due to concerns of imprecision, and heterogeneity (Supporting Information S1: Supplement S8).

**TABLE 2:** Baseline characteristics of patients.

Study ID	Participants	Age (mean ± SD)	Female (%)	Race (white) (%)	Weight (kg) (mean, SD)	BMI (kg/m^2^) (mean ± SD)	Binge episodes/week (mean ± SD)	Binge days/week (mean ± SD)	Most common adverse event (% of participants)
McElroy, Hudson, Mitchell, et al. (2015)	260	38.7 ± 10.2	81.5%	78%	98.6 ± 17.8	34.9 ± 5.3	5.6 ± 2.6	4.5 ± 1.3	Dry mouth (29%)
McElroy, Hudson, Ferreira‐Cornwell, et al. (2015)	383	38.1 ± 10.3	86.5%	77.6%	93.5 ± 19.5	33.5 ± 6.3	6.2 ± 2.8	4.7 ± 1.2	Dry mouth (24%)
McElroy, Hudson, Ferreira‐Cornwell, et al. (2015)	390	37.9 ± 10	85.2%	72.9%	93.9 ± 21	33.5 ± 6.3	6.5 ± 3.6	4.8 ± 1.4	Dry mouth (19%)
Guerdjikova et al. (2016)	50	37.7 ± 8.9	92%	78%	111.3 ± 26.4	39.8 ± 9.3	5.1 ± 3.1	4.2 ± 1.2	Dry mouth (24%)
Grilo et al. (2024)	61	44.3 ± 10.9	83.6%	75.4%	101.7 ± 17.9	36.1 ± 4.5	0.6 ± 1.1^a^	NR	Decreased appetite (23%)
Grilo et al. (2021)	22	50.4 ± 8.8	86.4%	72.7%	NR	37.1 ± 5.9	16.1 ± 11.9^a^	NR	Nausea (33%)
Grilo et al. (2022)	136	46.5 ± 12.2	81.6%	77.9%	102.9 ± 15.2	37.1 ± 4.9	17.6 ± 17.9^a^	NR	NR
Grilo, Lydecker, Jastreboff, et al. (2023)	89	45.7 ± 13.5	70.8%	69.7%	100.4 ± 22	35.3 ± 6.1	15.7 ± 11.2^a^	NR	NR
Grilo, Lydecker, Jastreboff, et al. (2023)	66	49.9 ± 12.1	84.8%	71.2%	97.8 ± 19	34.9 ± 5.1	0.9 ± 1.9^a^	NR	Constipation (23%)
McElroy et al. (2003)	61	40.8 ± 8.6	86.9%	NR	121.9 ± 21.7	44.2 ± 7.1	5.8 ± 2.8	4.6 ± 1.8	Paraesthesias (39%)
McElroy et al. (2007)	407	44.5 ± 11.5	84.2%	78.5%	106.5 ± 18.4	38.5 ± 5.3	6.5 ± 4.1	4.6 ± 1.3	Paraesthesias (34%)
Claudino et al. (2007)	73	38.3 ± 10.6	95.9%	57.5%	97.5 ± 14.1	37.4 ± 4.2	4.3 ± 2.6	3.8 ± 2.6	Paraesthesias (30%)

*Note:* The table summarises the baseline characteristics of patients included in the study. Characteristics include the number of participants, age, female participants, Race (White), weight, body mass index (BMI), Binge episodes/week, Binge days/week, Most Common Adverse Event. Mean values with SD are provided for continuous variables, while percentages are provided for categorical variables.

Abbreviations: BMI: Body Mass Index, BE: Binge Eating, NR: Not Reported.

^a^ Mean and standard deviation (SD) for binge episodes over a 28‐day period, rather than on a weekly basis.

### 3.2 Binge Episodes (Frequency)

**FIGURE 3:**
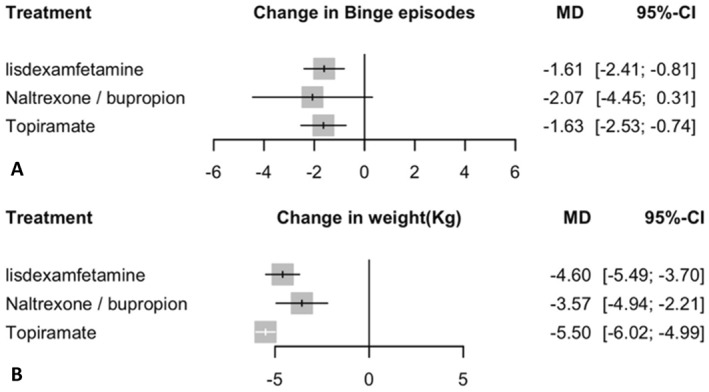
Results of binge episodes (frequency) and weight change. This figure presents a series of forest plots comparing the efficacy of lisdexamfetamine, naltrexone/bupropion, and topiramate against placebo in patients with BED. The outcomes measured are: (A) Binge episodes: The mean difference (MD) with 95% CI indicates the change in binge episodes (frequency) for each treatment compared to placebo. (B) Weight change: The mean difference (MD) with 95% CI indicates the weight change for each treatment compared to placebo.

In the random effects model assessing the change in Binge episodes (frequency) Lisdexamfetamine and topiramate are significantly reduced binge eating episodes (frequency) compared to placebo (Figure 3), with mean differences of −1.61 (95% CI: −2.41 to −0.81; *p* < 0.0001) and −1.63 (95% CI: −2.53 to −0.74; *p* = 0.0003), respectively. Naltrexone/bupropion showed a non‐significant trend toward reduced binge episode frequency (MD = −2.07, 95% CI: −4.45 to 0.31; *p* = 0.0883), but the confidence interval included zero, indicating substantial uncertainty due to high variability across studies. The heterogeneity analysis revealed substantial variability among studies, indicated by an I^2^ of 95.6%. Tests of heterogeneity within designs yielded significant results (*Q* = 203.53, df = 9, *p* < 0.0001), suggesting inconsistency among the study outcomes. Consequently, meta‐regression and sensitivity analyses were conducted to explore potential sources of heterogeneity. Transitivity assumption was held, with no substantial baseline imbalances detected in sex or BMI, though a significant imbalance was detected for Age (*p* = 0.0029). Among all interventions, naltrexone/bupropion ranked highest based on netrank analysis (P‐score 0.74), followed by topiramate (P‐score: 0.63), and lisdexamfetamine (P‐score: 0.61). Placebo consistently ranked lowest (Supporting Information S1: Supplement S7). League table summarising pairwise comparisons and rankings for change in binge episodes is provided in Supporting Information S1: Supplement S6, and the corresponding network plot is shown in Supporting Information S1: Supplement S2.

### 3.3 Change in Weight

In the random effects model assessing change in weight (kg) topiramate showed the most significant reduction with mean difference (MD) of −5.5 compared to placebo (Figure 3) (95% CI: −6.1 to −4.99, *p* < 0.0001). Lisdexamfetamine also showed a significant reduction with an MD of −4.6 (95% CI: −5.5 to −3.7 *p* < 0.0001), followed by Naltrexone/bupropion with a MD of −3.6 (95% CI: −4.9 to −2.21 *p* < 0.0001). The heterogeneity analysis demonstrated mild heterogeneity, with an I^2^ of 17.6%. The tests for heterogeneity within designs (*Q* = 9.71, df = 8, *p* = 0.2860) were not significant, suggesting consistency among the study outcomes. Transitivity assumption was held, with no substantial baseline imbalances detected in sex or BMI, though a significant imbalance was detected for Age (*p* = 0.0118). Among all interventions, topiramate ranked highest based on netrank analysis (P‐score: 0.98), followed by lisdexamfetamine (P‐score: 0.64), and naltrexone/bupropion (P‐score: 0.37). Placebo consistently ranked lowest (Supporting Information S1: Supplement S7). League table summarising pairwise comparisons and rankings for change in weight is provided in Supporting Information S1: Supplement S6, and the corresponding network plot is shown in Supporting Information S1: Supplement S2.

### 3.4 Safety Outcomes

#### 3.4.1 Serious Adverse Events (SAEs)

**FIGURE 4:**
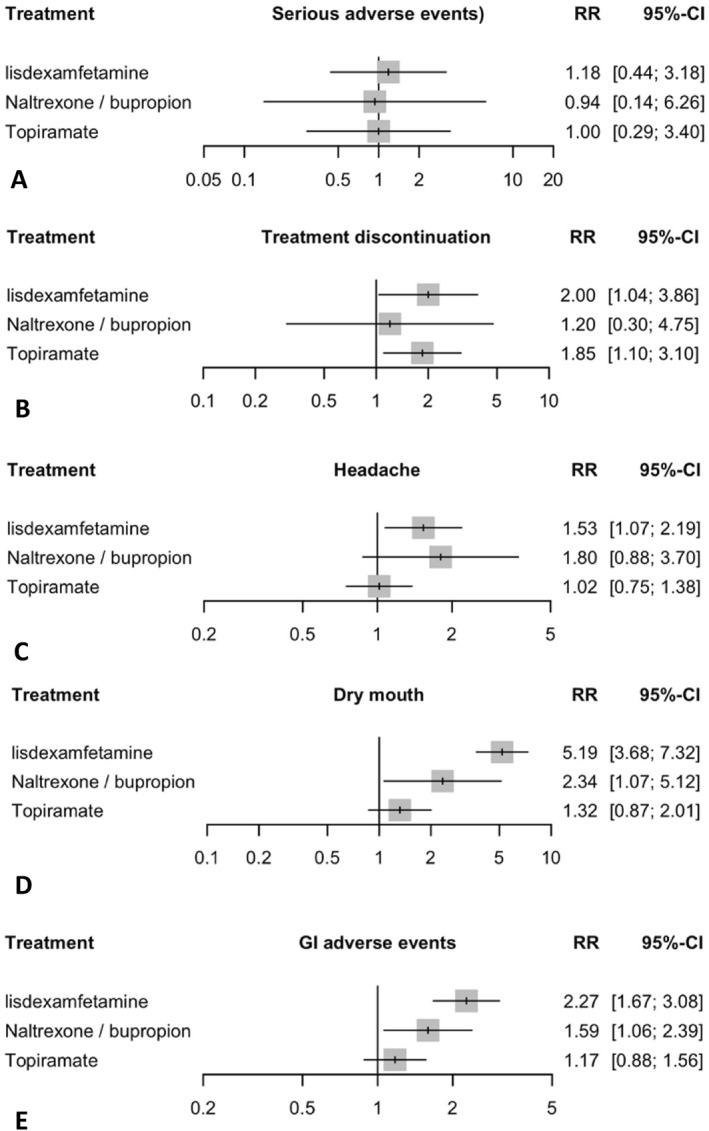
Safety outcomes. This figure presents a series of forest plots comparing the safety of lisdexamfetamine, naltrexone/bupropion, and topiramate treatments against placebo in patients with BED. The outcomes measured are (A) serious adverse events: The relative risk (RR) with 95% confidence intervals (CI) shows the likelihood of experiencing serious adverse events for each treatment compared to placebo. (B) Treatment discontinuation: The RR with 95% CI represents the likelihood of treatment discontinuation for each treatment compared to placebo. (C) Headache: The RR with 95% CI represents the likelihood of experiencing Headache for each treatment compared to placebo. (D) Dry mouth: The RR with 95% CI shows the likelihood of experiencing Dry mouth for each treatment compared to placebo. (E) GI adverse events The RR with 95% CI represents the likelihood of experiencing Dry mouth for each treatment compared to placebo. Grey squares represent effect estimates; horizontal lines show 95% CIs.

In terms of safety outcomes, A total of 10 studies were included in the main analysis for the prevalence of SAEs (Figure 4). In the random effects model assessing SAEs. All treatment groups showed a non‐significant increase in the risk of SAEs. The heterogeneity analysis demonstrated no significant heterogeneity, with an I^2^ = 0%. Tests for heterogeneity within designs were not significant (*Q* = 0.78, df = 7, *p* = 0.997), indicating consistency among the study results. Transitivity assumption was held, with no substantial baseline imbalances detected in sex or BMI, though a significant imbalance was detected for Age (*p* = 0.0059). The P‐scores, which rank treatments based on their effectiveness, with higher scores indicating more favourable outcomes, were highest for naltrexone/bupropion (0.544), followed by placebo (0.534), topiramate (0.521), and lisdexamfetamine (0.402) (Supporting Information S1: Supplement S7). League table summarising pairwise comparisons and rankings for SAEs is provided in Supporting Information S1: Supplement S6, and the corresponding network plot is shown in Supporting Information S1: Supplement S2.

#### 3.4.2 Treatment Discontinuation

In the random effects model assessing treatment discontinuation due to adverse events (Figure 4). Lisdexamfetamine had the highest discontinuation risk with relative risk (RR) of 2.00 (95% CI: 1.04 to 3.86, *p* = 0.0388) compared to placebo. Topiramate also showed high discontinuation rate with RR of 1.85 (95% CI: 1.11 to 3.10, *p* = 0.0193). Naltrexone/bupropion showed a non‐significant increase in the risk of discontinuation with RR of 1.20 (95% CI: 0.30 to 4.75, *p* = 0.7946). The heterogeneity analysis demonstrated no significant heterogeneity, with an I^2^ = 0%. Tests for heterogeneity within designs were not significant (*Q* = 2.33, df = 8, *p* = 0.9691), indicating consistency among the study outcomes. Transitivity assumption was held, with no substantial baseline imbalances detected in sex or BMI, though a significant imbalance was detected for Age (*p* = 0.0039). The P‐scores, which rank treatments based on their effectiveness, with higher scores indicating more favourable outcomes, were highest for Placebo (0.86), followed by Naltrexone/bupropion (0.62), topiramate (0.29), and lisdexamfetamine (0.23) (Supporting Information S1: Supplement S7). League table summarising pairwise comparisons and rankings for treatment discontinuation is provided in Supporting Information S1: Supplement S6, and the corresponding network plot is shown in Supporting Information S1: Supplement S2.

#### 3.4.3 Headache

In the random effects model assessing adverse events, lisdexamfetamine showed the highest significant increase in risk of headache with a relative risk (RR) of 1.54 (95% CI: 1.0750 to 2.1883, *p* = 0.0183) compared to placebo (Figure 4). Topiramate and Naltrexone/bupropion both showed a non‐significant increase in the risk of headache (RR 1.02; 95% CI: 0.751 to 1.38, *p* = 0.9111) and (RR 1.8; 95% CI: 0.86 to 3.7, *p* = 0.1102), respectively. The heterogeneity analysis demonstrated no significant heterogeneity, with an I2 = 0%. Tests for heterogeneity within designs were not significant (*Q* = 5.69, df = 7, *p* = 0.5763), indicating consistency among the study outcomes. Transitivity assumption was held, with no substantial baseline imbalances detected in sex or BMI, though a significant imbalance was detected for Age (*p* = 0.0059). The P‐scores, which rank treatments based on their effectiveness, with higher scores indicating more favourable outcomes, were highest for Placebo (0.83), followed by topiramate (0.78), lisdexamfetamine (0.24), and Naltrexone/bupropion (0.16). This ranking indicates that Naltrexone/bupropion had the highest risk of headache, followed by lisdexamfetamine, with topiramate showing the least increase in risk compared to placebo (Supporting Information S1: Supplement S7). League table summarising pairwise comparisons and rankings for headache is provided in Supporting Information S1: Supplement S6, and the corresponding network plot is shown in Supporting Information S1: Supplement S2.

#### 3.4.4 Dry Mouth

In the random effects model assessing adverse events. Lisdexamfetamine showed the highest significant increase in risk of dry mouth with a relative risk (RR) of 5.19 (95% CI: 3.6773 to 7.3155, *p* < 0.0001) compared to placebo (Figure 4). Naltrexone/bupropion followed with a significant increase in risk, presenting an RR of 2.34 (95% CI: 1.0679 to 5.1197, *p* = 0.0336). Topiramate showed a non‐significant increase in the risk of dry mouth with RR of 1.32 (95% CI: 0.8687 to 2.0058, *p* = 0.1934). The heterogeneity analysis demonstrated no significant heterogeneity, with an I2 = 0%. Tests for heterogeneity within designs were not significant (*Q* = 2.11, df = 6, *p* = 0.9092), indicating consistency among the study outcomes. Transitivity assumption was held, with no substantial baseline imbalances detected in sex or BMI, though a significant imbalance was detected for Age (*p* = 0.0065). The P‐scores, which rank treatments based on their effectiveness, with higher scores indicating more favourable outcomes, were highest for Placebo (0.96), followed by topiramate (0.66), naltrexone/bupropion (0.36), and lisdexamfetamine (0.01). This ranking indicates that lisdexamfetamine had the highest risk of dry mouth, followed by naltrexone/bupropion, with topiramate showing the least increase in risk compared to placebo (Supporting Information S1: Supplement S7). League table summarising pairwise comparisons and rankings for dry mouth is provided in Supporting Information S1: Supplement S6, and the corresponding network plot is shown in Supporting Information S1: Supplement S2.

#### 3.4.5 Gastrointestinal Adverse Events

In the random‐effects model assessing gastrointestinal (GI) adverse events—including nausea, vomiting, GI disturbances, decreased appetite, dyspepsia, diarrhoea, and/or constipation. Lisdexamfetamine showed the highest significant increase in risk of Gastrointestinal (GI) adverse events with a relative risk (RR) of 2.27 (95% CI: 1.6727 to 3.0756, *p* < 0.0001) compared to placebo (Figure 4). Naltrexone/bupropion followed with a significant increase in risk, presenting an RR of 1.59 (95% CI: 1.0614 to 2.3851, *p* = 0.0245). Topiramate showed a non‐significant increase in the risk of GI adverse events with RR of 1.17 (95% CI: 0.8831 to 1.56, *p* = 0.2698). The heterogeneity analysis demonstrated mild heterogeneity, with an I2 = 9.9%. Tests for heterogeneity within designs were not significant (*Q* = 7.77, df = 7, *p* = 0.3538), indicating consistency among the study outcomes. Transitivity assumption was held, with no substantial baseline imbalances detected in sex or BMI, though a significant imbalance was detected for Age (*p* = 0.0059). The P‐scores, which rank treatments based on their effectiveness, with higher scores indicating more favourable outcomes, were highest for Placebo (0.95), followed by topiramate (0.67), naltrexone/bupropion (0.35), and lisdexamfetamine (0.03). This ranking indicates that lisdexamfetamine had the highest risk of GI adverse events, followed by naltrexone/bupropion, with topiramate showing the least increase in risk compared to placebo (Supporting Information S1: Supplement S7). League table summarising pairwise comparisons and rankings for GI adverse events is provided in Supporting Information S1: Supplement S6, and the corresponding network plot is shown in Supporting Information S1: Supplement S2.

## 4 Discussion

The findings of this network meta‐analysis offer important clinical insights into the efficacy and safety of topiramate, lisdexamfetamine, and naltrexone/bupropion in treating BED. Topiramate and lisdexamfetamine effectively reduced binge episode (frequency), with pooled mean differences of −1.63 (95% CI: −2.53 to −0.74) and −1.61 (95% CI: −2.41 to −0.81), respectively, while also promoting weight loss. Naltrexone/bupropion significantly reduced weight, it didn't significantly affect binge frequency. Although topiramate and lisdexamfetamine significantly reduced binge episodes, the decrease of about 1.6 episodes per week may have only a modest impact for some patients, depending on how severe binge eating is at baseline. More research is needed to define what level of change is truly meaningful for patients.

Safety analysis showed that none of the drugs included in this network meta‐analysis had significant increase of serious adverse events, which is reassuring from a clinical perspective (Moawad et al. 2024; Coghill et al. 2014; Nourredine et al. 2021). However, both topiramate and lisdexamfetamine were associated with increased risk of discontinuation due to adverse events compared to placebo. Notably, lisdexamfetamine was associated with the highest risk of experiencing dry mouth and gastrointestinal (GI) adverse events, whereas topiramate demonstrated the lowest risk for these specific side effects (Brownley et al. 2016). This emphasises the significance of assessing tolerance and customising treatment according to specific individual needs and risk profiles.

The observed discrepancies in adverse events ranking (e.g., headache, treatment discontinuation) arise from differences in ranking metrics and the way uncertainty is incorporated in network meta‐analysis (Chiocchia et al. 2023). For example, lisdexamfetamine showed the highest risk for headache using relative risk estimates, while P‐score rankings, which account for both direct and indirect evidence plus uncertainty, sometimes ranked naltrexone/bupropion higher due to study variability. Similar differences were seen for treatment discontinuation, highlighting how analytic methods and data heterogeneity influence treatment hierarchies.

The observed differences in efficacy and safety among the drugs may be partially explained by their mechanism of actions. Topiramate modulates GABAergic and glutamatergic neurotransmission, which may reduce appetite and impulsive eating behaviours, contributing to both binge reduction and weight loss (Kaminski et al. 2004; Johnson et al. 2003). Lisdexamfetamine, a prodrug of d‐amphetamine, which increases dopamine, norepinephrine, and serotonin activity in the brain, causing appetite suppression, decrease food reward, and improve impulsivity, but often results in sympathomimetic side effects such as dry mouth, headache, and GI symptoms (Schneider et al. 2021). The Naltrexone/bupropion combination works by stimulating pro‐opiomelanocortin (POMC) neurons via bupropion and blocking inhibitory opioid feedback on POMC via naltrexone. This combined mechanism targets reward‐driven eating rather than total caloric intake, which may explain its stronger effect on weight than on binge behaviour (Billes and Greenway 2011; Cowley et al. 2001; Greenway et al. 2009).

Topiramate and lisdexamfetamine have both showed significant benefits in reducing binge‐eating episodes and promoting weight loss compared to placebo, as supported by multiple randomized controlled trials and systematic reviews (Brownley et al. 2016; Nourredine et al. 2021). In contrast, naltrexone/bupropion was also shown to significantly reduce the weight but not the binge frequency when compared to placebo as demonstrated in pervious RCTs (Grilo et al. 2021, 2022, 2023). These findings are consistent with a recent systematic review and network meta‐analysis, which also identified topiramate and lisdexamfetamine as the most effective agents for reducing binge‐eating episodes, while naltrexone/bupropion showed limited efficacy. Notably, lisdexamfetamine was associated with a higher risk of adverse events, particularly dry mouth and gastrointestinal (GI) symptoms, a result that was consistent with our analysis (Costa et al. 2025). By incorporating both direct and indirect evidence, this network meta‐analysis provides a comprehensive understanding on the efficacy and safety of these three treatments.

Our analysis showed that Pharmacological therapy including either topiramate, lisdexamphetamine or naltrexone/bupropion have all been shown to significantly reduce weight. This is especially important when comparing it to the first line treatment for binge eating disorder, CBT, which has not been shown to significantly affect weight (Wilfley et al. 2002; Grilo et al. 2005; Devlin et al. 2005) Thus, pharmacotherapy can enhance the benefits of CBT by promoting a clinically significant weight reduction and greater remission of binge eating in the short run (Claudino et al. 2007).

This study has several notable strengths. To increase the internal validity of our findings, we carried out a thorough literature search across major databases without language limitations and the majority of our included studies were randomized controlled trials. We were able to rank the therapies according to their efficacy and compare several of them at once by using a frequentist network meta‐analysis. We used robust methodological tools such as structured transitivity assessment using baseline covariate balance and the CINeMA framework to assess the certainty of the evidence. Our sensitivity analyses, which included leave‐one‐out testing and the removal of high‐risk studies (Supporting Information S1: Supplement S9), confirmed the robustness and consistency of our results.

Despite these strengths, our analysis has limitations. Variability across studies in populations, interventions, and outcome measures contributed to the considerable heterogeneity observed in binge episode analysis (*I* ^*2*^ = 95.6%, *Tau* ^*2*^ = 0.57). Which remained unexplained even after meta‐regression and sensitivity analyses (including exclusion of high‐risk studies and leave‐one‐out analysis). Variation in the methods used to measure binge eating episodes across trials (e.g., structured interviews such as the EDE, self‐reports, or food diaries) may also have contributed to this heterogeneity. Despite this, the network was found to be globally consistent based on design decomposition testing, with no statistically significant inconsistency detected between the studies. The transitivity assumption held, with no substantial baseline imbalances detected in sex or BMI, though a significant imbalance was detected for Age (*p* = 0.0029). Additional limitations include the exclusion of binge eating (BE) remission as an outcome, since it was inconsistently reported across trials and variably defined (ranging from 1 to 4 weeks free of binge episodes), which prevented systematic evaluation. Moreover, our analysis focused only on lisdexamfetamine, topiramate, and naltrexone/bupropion, excluding other pharmacological agents such as antidepressants that have been evaluated for BED, which may limit the comprehensiveness of our conclusions. The network was also anchored by placebo in all comparison, limiting the strength of indirect comparisons. Finally, publication bias could not be completely ruled out due to limited funnel plot asymmetry tests for small networks (Supporting Information S1: Supplement S5).

While our network meta‐analysis identifies promising pharmacologic strategies for BED, several gaps remain. Studies with longer follow‐up periods are also needed to assess the long‐term tolerability and efficacy for treatments. Furthermore, 58% of patients with BED have comorbid psychiatric conditions (Hambleton et al. 2022), the majority of original trials excluded those patients, and also excluded those with substance use disorder in order to enhance sample homogeneity. Further trials that include this population would enhance the generalisability and clinical applicability of future evidence. Finally, larger trials with expanded sample sizes, and using validated scales are needed to guide clinical practice for this highly prevalent disorder.

## 5 Conclusion

This network meta‐analysis found that topiramate and lisdexamfetamine appear to be more effective than naltrexone/bupropion or placebo in treating binge eating disorder, significantly reducing both binge episode (frequency) and weight. Although lisdexamfetamine was effective, it was associated with a higher risk of dry mouth and gastrointestinal adverse events. Naltrexone/bupropion significantly reduced weight but did not demonstrate consistent effects on binge frequency. Our findings provide actionable insight into the efficacy and safety of available pharmacologic options for BED and underscore the need for further head‐to‐head trials in specific patient populations.

Our findings are consistent with current guidelines for BED, which emphasise the importance of individualised, integrative care. Pharmacologic agents like lisdexamfetamine and topiramate effectively reduce binge frequency and assist weight management, particularly in individual refractory to psychotherapy or preferring medication. However, guidelines recommend that medication be combined with evidence‐based psychotherapies, including cognitive behavioural therapy, interpersonal psychotherapy, and Behavioural weight loss interventions, which address the underlying psychological mechanisms of BED. Incorporating nutritional counselling and psychosocial support within a multidisciplinary framework further address both behavioural and metabolic aspects of the disorder. Such an approach enables treatment to be tailored to individual symptom profiles, comorbid conditions, and individual preferences, thereby improving long‐term adherence and overall quality of life in individuals with BED.

## Author Contributions

**Tamer Hodrob:** conceptualization, methodology, writing – original draft, writing – review and editing, formal analysis, project administration, investigation. **Ibrahim Ismail:** conceptualization, methodology, investigation, writing – original draft, writing – review and editing, project administration. **Alaaeddin Abusalameh:** methodology, conceptualization, project administration, and review of final draft. **Celina R. Andonie:** methodology, review of final draft, conceptualization, and project administration. **Omar Ayesh:** review of final draft, formal analysis. **Hazem Ayesh:** review of final draft, conceptualization, formal analysis, and project administration. All authors have read and agreed to the published version of the manuscript.

## Ethics Statement

The authors have nothing to report.

## Consent

The authors have nothing to report.

## Conflicts of Interest

The authors declare no conflicts of interest.

## Supporting information


Supporting Information S1

## Data Availability

The data supporting the findings of this study are available from the corresponding author upon reasonable request.
