# Supplementary material for: Efficacy and Safety of Lisdexamfetamine Versus Topiramate Versus Naltrexone/Bupropion in Individuals With Binge Eating Disorder: A Network Meta‐Analysis
Source: Eur Eat Disord Rev. 2025 Sep 22;34(2):334–46. doi: 10.1002/erv.70035 (PMC12862547; doi:10.1002/erv.70035)
Supplement: Supplementary file 1 — Supporting Information S1 [file ERV-34-334-s001.docx]

Table of Contents

*Supplement S1. Search strategy4*

*Supplement S2. Network Plot of Treatment Comparisons 5*

Figure S2.1: Network Plot of Treatment Comparisons for binge eating episodes (frequency)*5*

Figure S2.2. Network Plot of Treatment Comparisons for weight 6

Figure S2.3. Network Plot of Treatment Comparisons for serious adverse events7

Figure S2.4. Network Plot of Treatment Comparisons treatment discontinuation 8

Figure S2.5. Network Plot of Treatment Comparisons for headache 9

Figure S2.6. Network Plot of Treatment Comparisons for dry mouth 10

Figure S2.7. Network Plot of Treatment Comparisons for gastrointestinal adverse events11

*Supplement S3. Baseline characteristics of the included studies12*

Table S3: Study Characteristics and Outcomes in the included Clinical Trials13

*Supplement S4. Risk of bias assessment of included trials18*

Table S4: Risk of bias assessment18

*Supplement S5. Publication bias (funnel plot)20*

Figure S5.1. Binge episodes (frequency)20

Figure S5.2. Weight21

Figure S5.3. Serious adverse events21

Figure S5.4 Treatment discontinuation22

Figure S5.5 Headache22

Figure S5.6. Dry mouth23

Figure S5.7. Gastrointestinal adverse events23

*Supplement S6. League table for each outcome24*

Table S6.1. Binge episodes (frequency)24

Table S6.2. Weight24

Table S6.3. Serious adverse events24

Table S6.4 Treatment discontinuation25

Table S6.5 Headache25

Table S6.6. Dry mouth25

Table S6.7. Gastrointestinal adverse events26

*Supplement S7. Treatment ranking for each outcome27*

Table S7.1. Binge episodes (frequency)27

Table S7.2. Weight27

Table S7.3. Serious adverse events27

Table S7.4 Treatment discontinuation28

Table S7.5 Headache28

Table S7.6. Dry mouth28

Table S7.7. Gastrointestinal adverse events29

*Supplement S8. Certainty of evidence for each outcome30*

Table S8.1. Binge episodes (frequency)30

Table S8.2. Weight31

Table S8.3. Serious adverse events32

Table S8.4 Treatment discontinuation33

Table S8.5 Headache34

Table S8.6. Dry mouth35

Table S8.7. Gastrointestinal adverse events36

*Supplement S9. Sensitivity analysis37*

Figure S9.1.1 Binge episodes (frequency) for large sample studies. Network geometry 37

Figure S9.1.2 Binge episodes (frequency) for large sample studies. Funnel plot 38

Figure S9.1.3 Binge episodes (frequency) for large sample studies. Forest plot 38

Figure S9.2.1 Binge episodes (frequency) for low-risk studies. Network geometry 39

Figure S9.2.2 Binge episodes (frequency) for low-risk studies. Funnel plot40

Figure S9.2.3 Binge episodes (frequency) for large sample studies. Forest plot40

Figure S9.3.1 Weight for large sample studies. Network geometry41

Figure S9.3.2 Weight for large sample studies. Funnel plot42

Figure S9.3.3 Weight for large sample studies. Forest plot42

Figure S9.4.1 Weight for low-risk studies. Network geometry43

Figure S9.4.2 Weight for low-risk studies. Funnel plot44

Figure S9.4.3 Weight for low-risk studies. Forest plot44

Table S9.5.1 leave one out analysis for binge episodes (frequency) 45

Table S9.5.2 leave one out analysis for Weight46

Table S9.5.3 leave one out analysis for Serious adverse events47

Table S9.5.4 leave one out analysis for Treatment discontinuation48

Table S9.5.5 leave one out analysis for Headache49

Table S9.5.6 leave one out analysis for Dry mouth50

Table S9.5.7 leave one out analysis for Gastrointestinal adverse events51

*Supplement S10. PRISMA check list52*

**Supplement S1. Search strategy**

From inception to February 08, 2025:

****PubMed search Syntax: ****

(("Binge Eating Disorder" OR BED OR "Eating Disorder")  AND ("Lisdexamfetamine" OR "Vyvanse" OR "Topiramate" OR "Topamax" OR "Trokendi XR" OR "Qudexy XR" OR "Naltrexone" OR "ReVia" OR "Vivitrol" OR "Depade" OR "Bupropion" OR "Wellbutrin" OR "Wellbutrin SR" OR "Wellbutrin XL" OR "Aplenzin" OR "Forfivo XL" OR "Zyban" OR "Contrave")  AND ("randomized controlled trial" OR RCT OR "clinical trial" OR "trial")  AND ("binge eating episodes" OR "binge" OR "binge frequency" OR "eating frequency" OR "weight loss" OR "weight management" OR "weight" OR "BMI" OR "treatment outcomes" OR "adverse events" OR "safety profile"))

****** **Scopus search Syntax: ****

(("Binge Eating Disorder" OR BED OR "Eating Disorder")  AND ("Lisdexamfetamine" OR "Vyvanse" OR "Topiramate" OR "Topamax" OR "Trokendi XR" OR "Qudexy XR" OR "Naltrexone" OR "ReVia" OR "Vivitrol" OR "Depade" OR "Bupropion" OR "Wellbutrin" OR "Wellbutrin SR" OR "Wellbutrin XL" OR "Aplenzin" OR "Forfivo XL" OR "Zyban" OR "Contrave")  AND ("randomized controlled trial" OR RCT OR "clinical trial" OR "trial")  AND ("binge eating episodes" OR "binge" OR "binge frequency" OR "eating frequency" OR "weight loss" OR "weight management" OR "weight" OR "BMI" OR "treatment outcomes" OR "adverse events" OR "safety profile"))

****** **Cochrane Library search Syntax: ****

(("Binge Eating Disorder" OR BED OR "Eating Disorder")  AND ("Lisdexamfetamine" OR "Vyvanse" OR "Topiramate" OR "Topamax" OR "Trokendi XR" OR "Qudexy XR" OR "Naltrexone" OR "ReVia" OR "Vivitrol" OR "Depade" OR "Bupropion" OR "Wellbutrin" OR "Wellbutrin SR" OR "Wellbutrin XL" OR "Aplenzin" OR "Forfivo XL" OR "Zyban" OR "Contrave")  AND ("randomized controlled trial" OR RCT OR "clinical trial" OR "trial")  AND ("binge eating episodes" OR "binge" OR "binge frequency" OR "eating frequency" OR "weight loss" OR "weight management" OR "weight" OR "BMI" OR "treatment outcomes" OR "adverse events" OR "safety profile"))

****** **ClinicalTrials.gov search Syntax: ****

(("Binge Eating Disorder" OR BED OR "Eating Disorder")  AND ("Lisdexamfetamine" OR "Vyvanse" OR "Topiramate" OR "Topamax" OR "Trokendi XR" OR "Qudexy XR" OR "Naltrexone" OR "ReVia" OR "Vivitrol" OR "Depade" OR "Bupropion" OR "Wellbutrin" OR "Wellbutrin SR" OR "Wellbutrin XL" OR "Aplenzin" OR "Forfivo XL" OR "Zyban" OR "Contrave")  AND ("randomized controlled trial" OR RCT OR "clinical trial" OR "trial")  AND ("binge eating episodes" OR "binge" OR "binge frequency" OR "eating frequency" OR "weight loss" OR "weight management" OR "weight" OR "BMI" OR "treatment outcomes" OR "adverse events" OR "safety profile"

**Supplement S2: Network plots of treatment comparisons**

Figure S2.1: Network Plot of Treatment Comparisons for binge eating episodes (frequency)


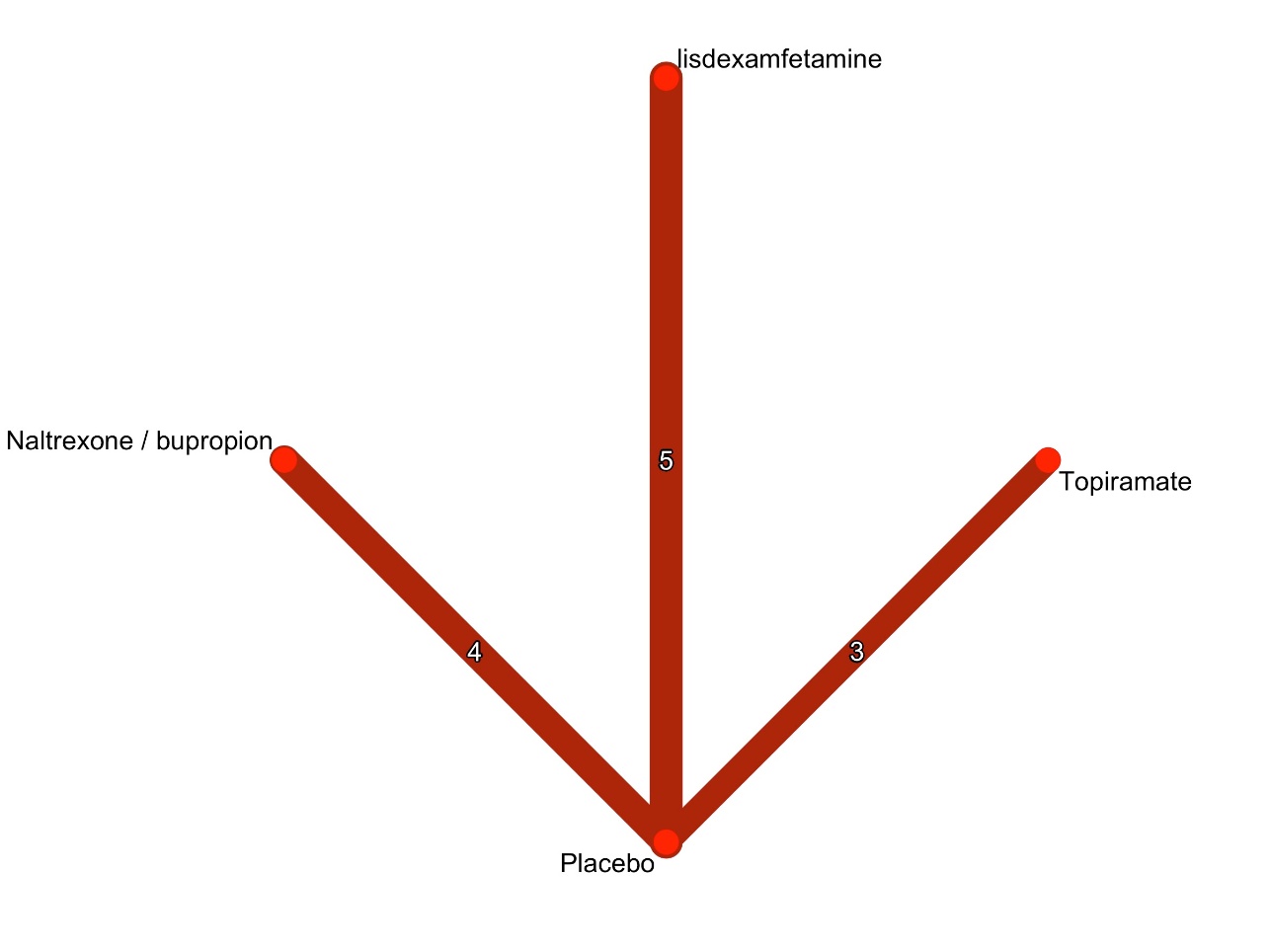


FigureS2.2: Network Plot of Treatment Comparisons for change in weight


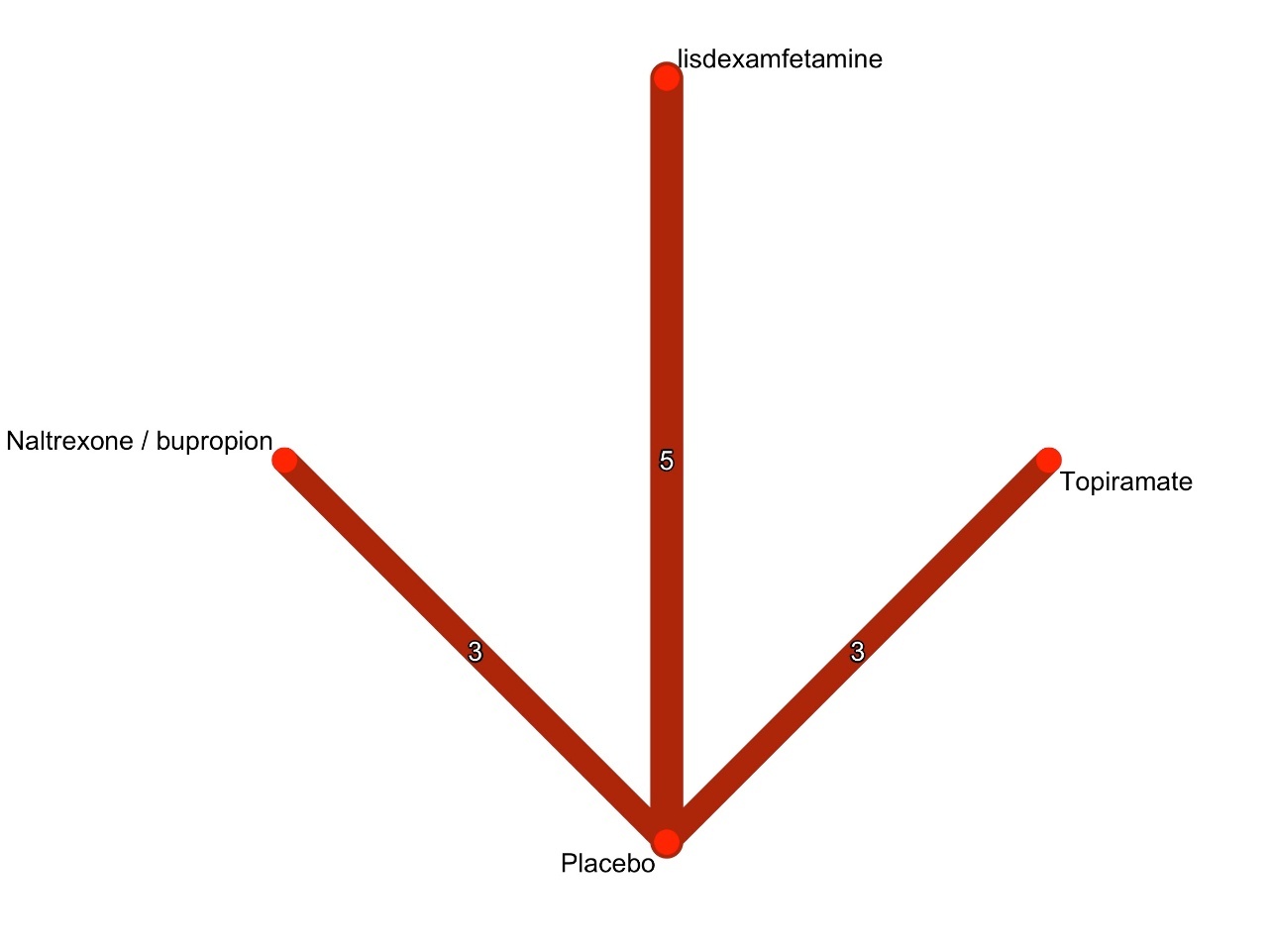


Figure S2.3 Network Plot of Treatment Comparisons for serious adverse events


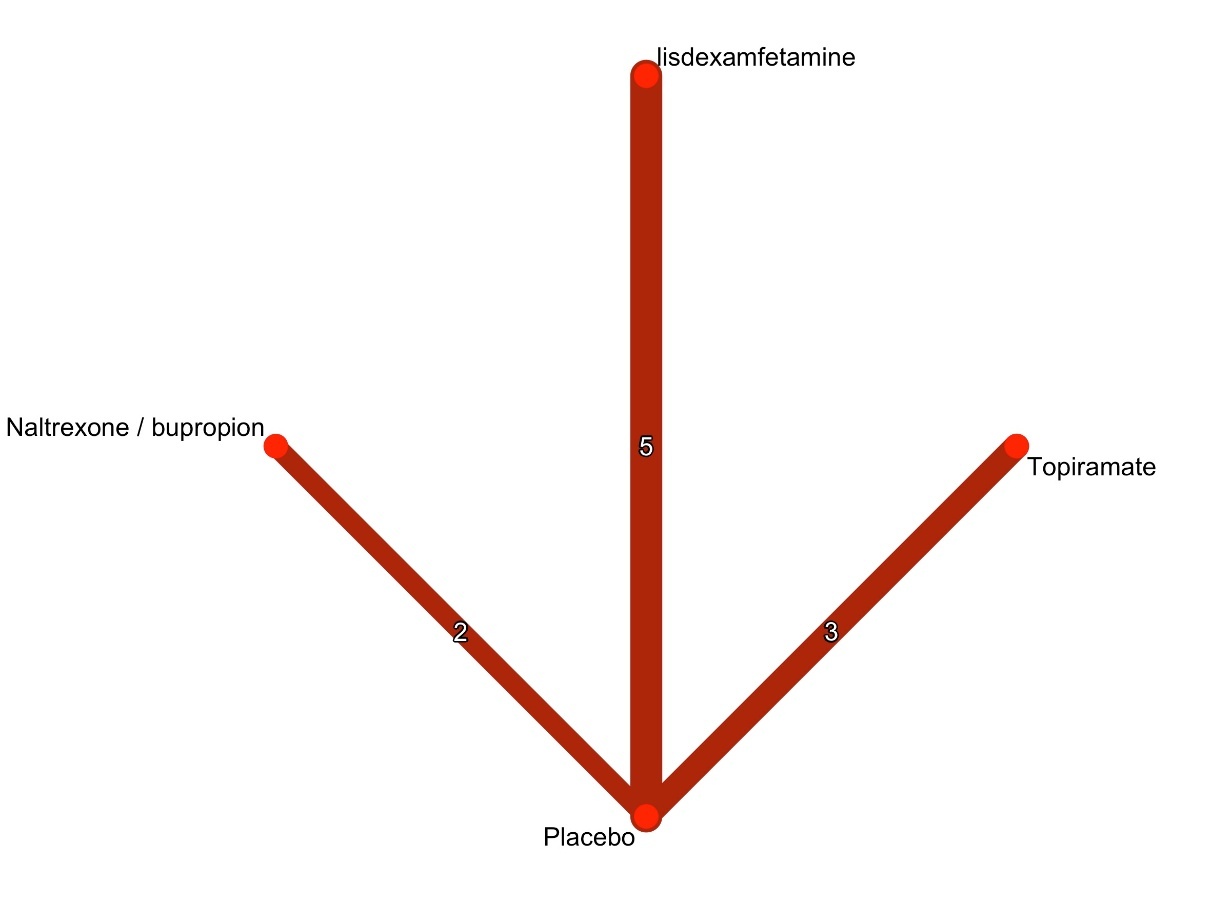


Figure S2.4 Network Plot of Treatment Comparisons treatment discontinuation


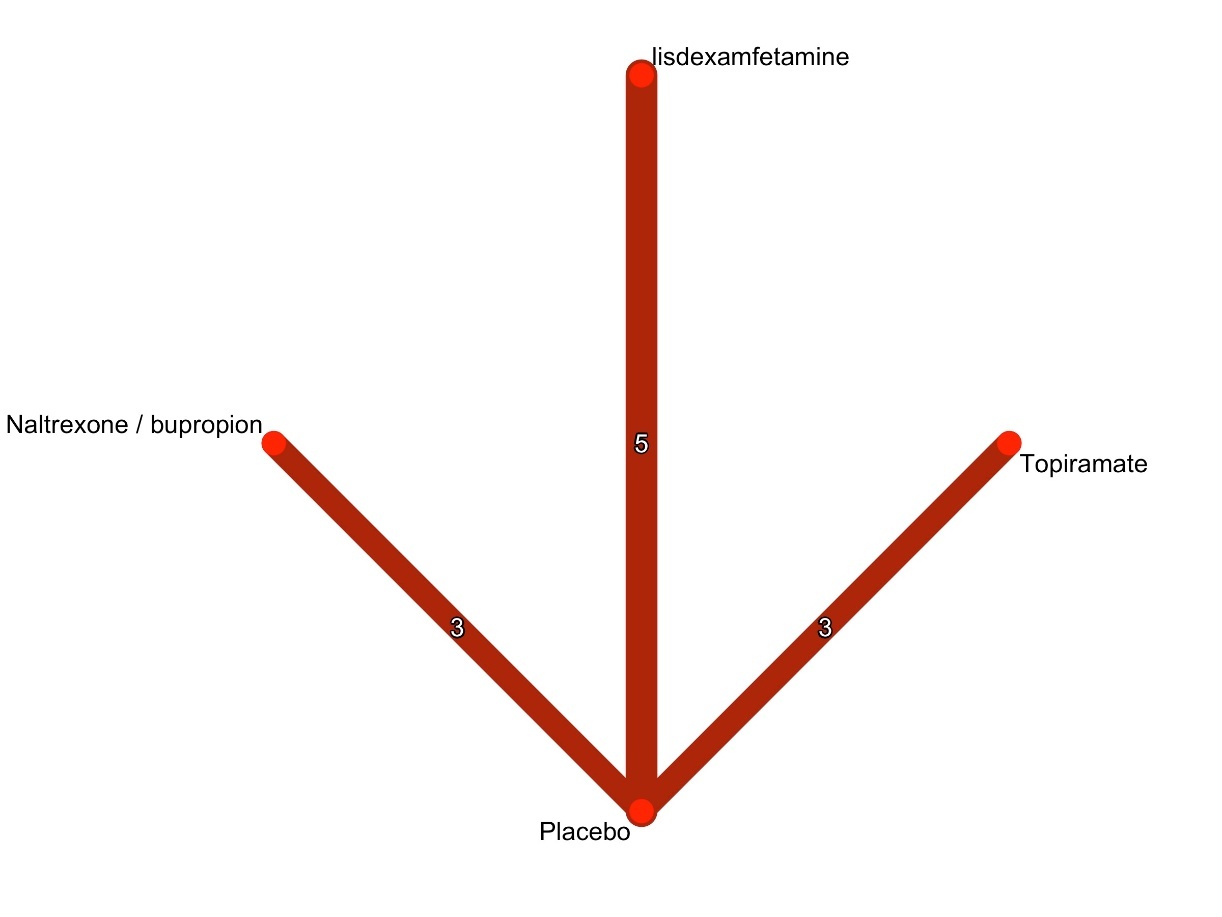


Figure S2.5 Network Plot of Treatment Comparisons for headache


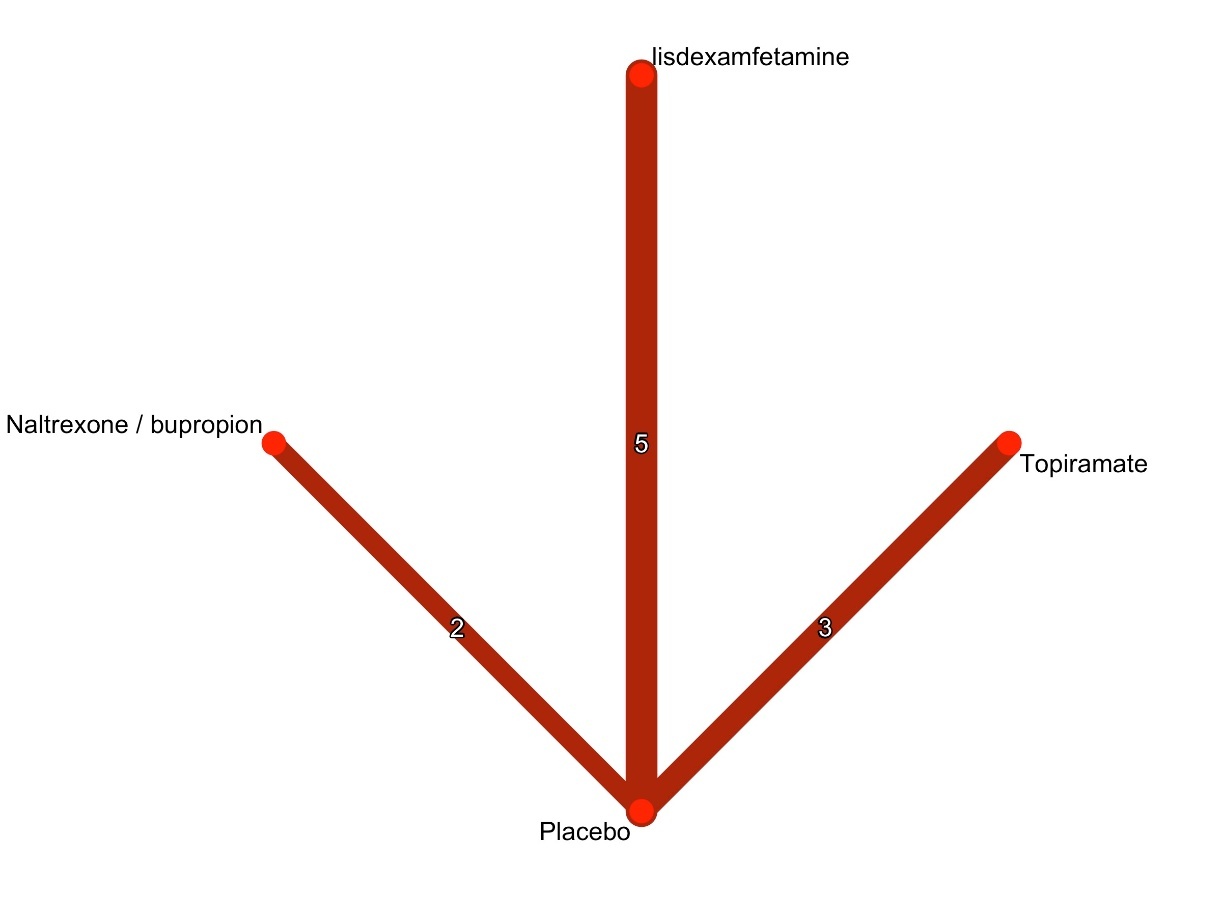


Figure S2.6. Network Plot of Treatment Comparisons for dry mouth


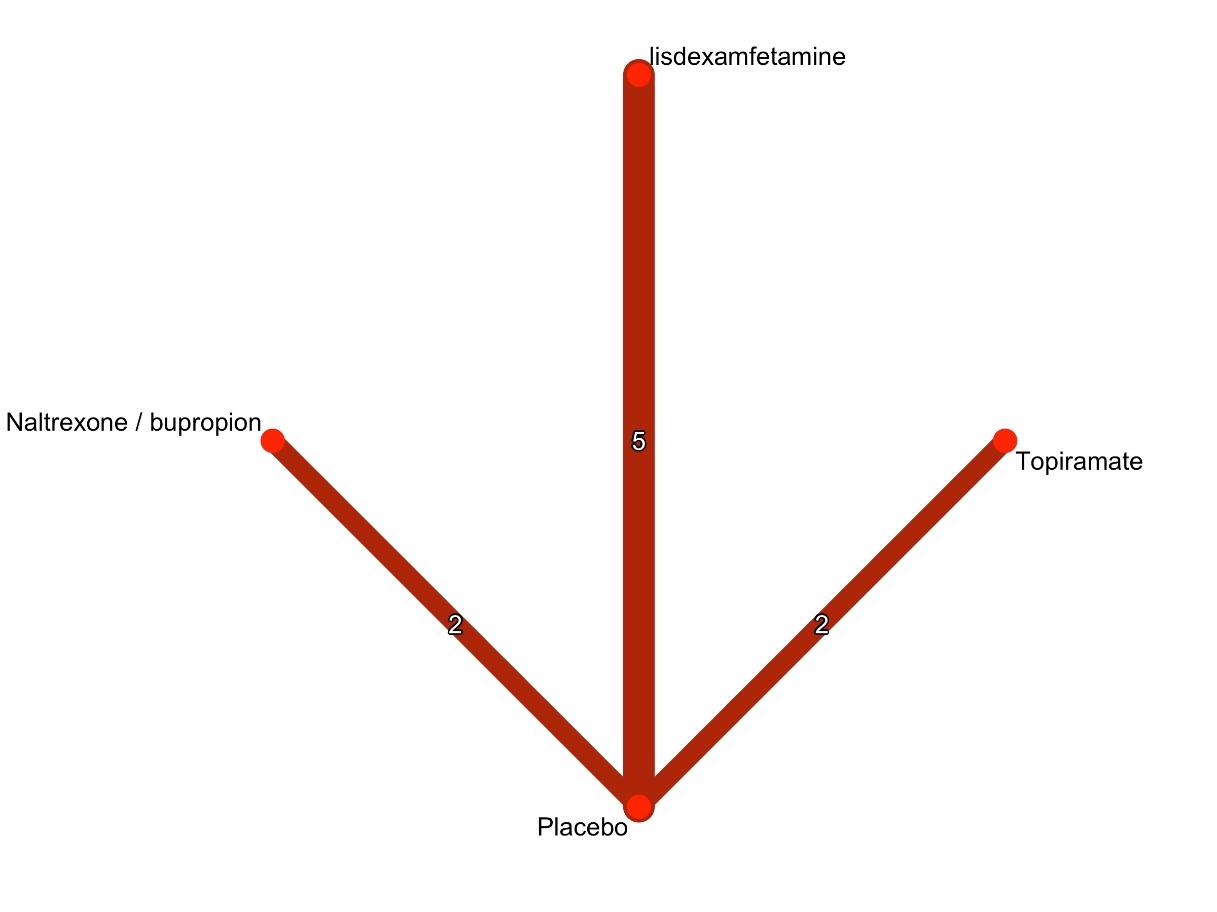


Figure S2.7 Network Plot of Treatment Comparisons for gastrointestinal adverse events


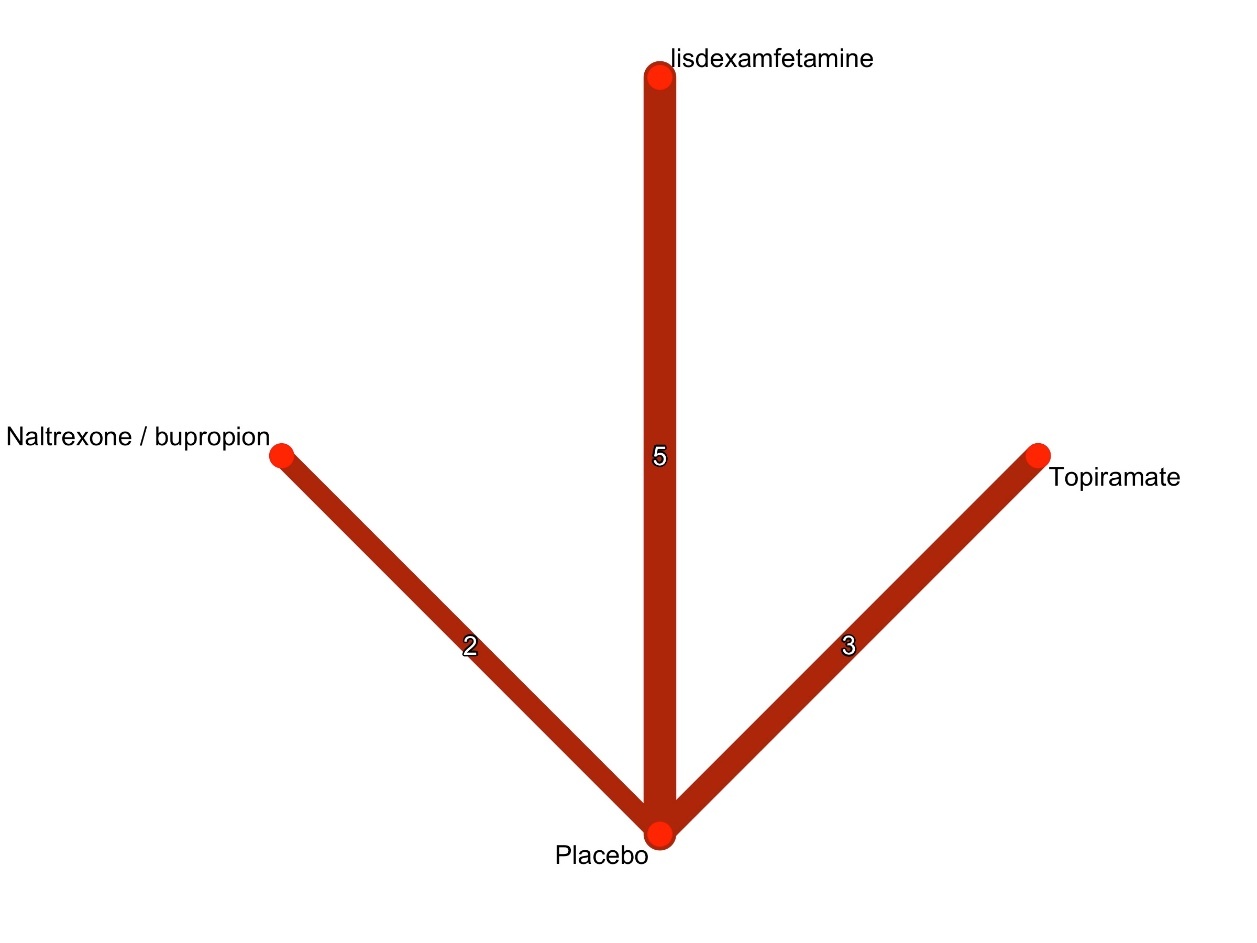


**Supplement S3**. Baseline characteristics of the included studies

Table S3: Study Characteristics and Outcomes in the included Clinical Trials

The table summarizes various BED clinical trials, detailing study design, registration, duration, treatment arms, primary and secondary outcomes, and population characteristics. Abbreviations used include RCT (randomized controlled trial), DB (double-blind), PC (placebo-controlled), BED (binge eating disorder).

| Study | Design | Registration | Duration | Treatment Arms | Primary Outcomes | Secondary Outcomes | Population | Population Excluded |
| --- | --- | --- | --- | --- | --- | --- | --- | --- |
| McElroy,2015 | Phase 2, RCT, DB, PC | NCT01291173 | 11 weeks | lisdexamfetamine (30 mg, 50 mg, 70 mg daily), placebo | Change in number of BE days/week | Change in number of BE episodes/week, weight change, CGI-I, self-reported Binge Eating Scale, Y-BOCS-BE, safety | BED, aged 18-55 years, BMI (25 - 45) | Excludes those with eating disorders (except BED), ADHD, psychiatric/cardiovascular conditions, recent psychological or weight-loss interventions (≤3 months), stimulant use (≤6 months), substance abuse, or recent/current drug use (30–60 days). |
| McElroy,2016_a1 | Phase 3, RCT, DB, PC | NCT01718483 | 11-12 weeks | lisdexamfetamine (30 mg-70 mg daily), placebo | Change in number of BE days/week | Change in CGI-I, 4-week binge eating cessation, Y-BOCS-BE, percentage weight, fasting triglyceride, safety | BED, aged 18-55 years, ≥3 BE days/week for 2 weeks, BMI (18 - 45) | Excludes those with eating disorders (except BED), ADHD, psychiatric disorders, suicide attempts, cardiovascular conditions, recent weight-loss interventions (≤3 months), stimulant use (≤6 months), substance abuse (except nicotine), or LDX allergy. |
| McElroy,2016_a2 | Phase 3, RCT, DB, PC | NCT01718509 | 11-12 weeks | lisdexamfetamine (30 mg-70 mg daily), placebo | Change in number of BE days/week | Change in CGI-I, 4-week binge eating cessation, Y-BOCS-BE, percentage weight, fasting triglyceride, safety | BED, aged 18-55 years, ≥3 BE days/week for 2 weeks, BMI (18 - 45) | Excludes those with eating disorders (except BED), ADHD, psychiatric disorders, suicide attempts, cardiovascular conditions, recent weight-loss interventions (≤3 months), stimulant use (≤6 months), substance abuse (except nicotine), or LDX allergy. |
| Guerdjikova,2016 | Phase 3, RCT, DB, PC | NCT01090713 | 12 weeks | lisdexamfetamine (30 mg-70 mg daily), placebo | Change in number of BE days/week | Change in number of BE episodes/week, CGI-I, Y-BOCS-BE, weight, BMI, FCI scale, metabolic variables, safety | BED, aged 18-55 years, ≥3 BE days/week for 2 weeks, BMI (18 - 45) | Excludes those with eating disorders (except BED), ADHD, psychiatric disorders, suicide attempts, cardiovascular conditions, recent weight-loss interventions (≤3 months), stimulant use (≤6 months), substance abuse (except nicotine), or LDX allergy, and pregnancy, or lactation. |
| Grilo,2024 | Phase 3, RCT, DB, PC | NCT03926052 | 12 weeks | lisdexamfetamine (30 mg-70 mg daily), placebo | Change in EDE BE (BE frequency) | Change in EDE-Q BE, Weight, EDE Global Score, BDI-II Depression Score, safety | BED, aged 18-64 years, ≥65% reduction in binge-eating after CBT and LDX (Stage 1 responders), BMI (27 - 50) | Excludes those with eating disorders (except BED), recent weight-loss interventions psychiatric/cardiovascular conditions, trial participation, substance abuse, seizures, or pregnancy/breastfeeding. |
| Grilo,2021 | NR, pilot RCT, DB, PC | NCT02317744 | 26 weeks | Naltrexone/Bupropion XL 50/300 mg daily, placebo | Change in BE frequency | Change in global eating-disorder psychopathology, Percentage weight, BMI, BDI-II Depression Score, safety | BED, aged 18-65 years, BMI (30 - 50) | Excludes those with eating disorders (except BED), recent eating/weight-related treatment, psychiatric/cardiovascular conditions, NB contraindications/allergy, use of contraindicated medications, or pregnancy/breastfeeding. |
| Grilo,2022 | Phase 2 Phase 3, RCT, DB, PC | NCT03045341 | 16 weeks | Naltrexone/Bupropion SR 32/360 mg daily, BWL, placebo | Change in EDE BE (BE frequency), weight | Change in EDE-Q BE, EDE Global Score, BDI-II Depression Score, FCI Score, PFS Score, TFEQ Restraint Score, TFEQ Disinhibition Score, metabolic variables | BED, aged 18-70 years, BMI (27 - 50) | Excludes those with eating disorders (except BED), recent eating/weight-related treatment, psychiatric/cardiovascular conditions, NB contraindications/allergy, use of contraindicated medications, or pregnancy/breastfeeding |
| Grilo,2023_a | Phase 2 Phase 3, RCT, DB, PC | NCT03539900 | 12 weeks | Naltrexone/Bupropion SR 32/360 mg daily, placebo | Change in EDE BE (BE frequency), weight, BMI | Change in EDE Global Score, BDI-II Depression Score, FCI Score, PFS Score, TFEQ Restraint Score, TFEQ Disinhibition Score, metabolic variables | BED, aged 18-70 years, BMI (27 - 50), | Excludes those with eating disorders (except BED), recent eating/weight-related treatment, psychiatric/cardiovascular conditions, NB contraindications/allergy, use of contraindicated medications, or pregnancy/breastfeeding |
| Grilo,2023_b | Phase 3, RCT, DB, PC | NCT03047005 | 16 weeks | Naltrexone/Bupropion SR 32/360 mg daily, placebo | Change in BE frequency, weight | Change in EDE Global Score, BDI-II Depression Score, FCI Score, PFS Score, TFEQ Restraint Score, TFEQ Disinhibition Score, metabolic variables, safety | BED, aged 18-70 years, ≥65% reduction in binge-eating after NB or NB and BWL (Stage 1 responders), BMI (21.5 - 50), | Excludes those with eating disorders (except BED), recent eating/weight-related treatment, psychiatric/cardiovascular conditions, NB contraindications/allergy, use of contraindicated medications, or pregnancy/breastfeeding |
| McElroy,2003 | Phase 3, RCT, DB, PC | NR | 14 weeks | Topiramate 25-600 mg daily, placebo | Change in number of BE episodes/week | Change in number of BE days/week, weight change, CGI-I, Hamilton Depression Rating Scale, BMI, Y-BOCS-BE, safety | BED, aged 18-60 years, Y-BOCS-BE score ≥ 15, BMI ≥ 30 | Excludes those with recent (≤6 months) substance use disorder, psychiatric disorders, suicidality, unstable medical conditions, nephrolithiasis, seizures, abnormal labs, medication interactions with topiramate, or prior topiramate use. |
| McElroy,2007 | Phase 2 Phase 3, RCT, DB, PC | NCT00210808 | 16 weeks | Topiramate 25-400 mg daily, placebo | Change in number of BE days/week | Change in number of BE episodes/week, weight, BMI, CGI-I, Hamilton anxiety Rating Scale, TFEQ Disinhibition and Restraint, Barratt Impulsiveness Scale, Sheehan Disability Scale, Y-BOCS-BE, Montgomery-Åsberg Depression Rating Scale, safety | BED, aged 18-65 years, ≥3 BE days/week for 2 weeks, BMI (30 - 50) | Excludes individuals with substance use disorder (≤3 months), psychiatric/personality disorders, unstable or serious medical conditions, nephrolithiasis, seizures, topiramate contraindications/allergy, or pregnancy/breastfeeding. |
| Claudino,2007 | Phase 3, RCT, DB, PC | NCT00307619 | 21 weeks | Topiramate 25-200 mg daily, CBT, placebo | Change in weight | Change in number of BE episodes/week, number of BE days/week, BMI, BES scores, BDI-II Depression Score, safety | BED, aged 18-60 years, BES score > 17, BMI ≥ 30 | Excludes those with recent (≤3 months) substance use, weight-loss therapy, psychiatric disorders, suicidality, unstable medical conditions, nephrolithiasis, seizures, weight-loss surgery, topiramate topiramate contraindications/allergy, or pregnancy/breastfeeding. |

**Abbreviations:**

- RCT: Randomized Controlled Trial
- DB: Double-Blind
- PC: Placebo-Controlled
- BE: Binge Eating
- BED: Binge Eating Disorder
- CGI-I: Clinical Global Impressions–Improvement
- Y-BOCS-BE: Yale-Brown Obsessive Compulsive Scale modified for Binge Eating
- BMI: Body Mass Index
- FCI: Food Craving Inventory
- EDE: Eating Disorder Examination
- EDE-Q BE: Eating Disorder Examination- Questionnaire Binge Eating
- BDI-II: Beck Depression Inventory-II
- CBT: Cognitive Behavioral Therapy
- LDX: lisdexamfetamine
- PFS: Power of Food Scale
- TFEQ: Three Factor Eating Questionnaire
- NB: Naltrexone/Bupropion
- BWL: Behavioral Weight Loss
- BES: Binge Eating Scale

**Supplement S4.** Risk of bias assessment of included trials

| **Study ID** | **Random Sequence Generation** | **Allocation Concealment** | **Blinding of Participants/Personnel** | **Blinding of Outcome Assessment** | **Incomplete Outcome Data** | **Selective Reporting** | **Other Bias** | **Overall Judgment** |
| --- | --- | --- | --- | --- | --- | --- | --- | --- |
| McElroy,2015 | \| Low Risk \| \| --- \| | Low Risk | Low Risk | Low Risk | Low Risk | Low Risk | Some Concerns | Some Concerns |
| McElroy,2016_a1 | Low Risk | Low Risk | Low Risk | Some concerns | Some concerns | Some concerns | Some concerns | Some concerns |
| McElroy,2016_a2 | Low Risk | Low Risk | Low Risk | Some concerns | Some concerns | Some concerns | Some concerns | Some concerns |
| Guerdjikova,2016 | Low Risk | Low Risk | Low Risk | Low Risk | Low Risk | Some concerns | Some concerns | Some concerns |
| Grilo,2024 | Low Risk | Some concerns | Low Risk | Low Risk | Low Risk | Low Risk | Some concerns | Some concerns |
| Grilo,2021 | Low Risk | Low Risk | Low Risk | Low Risk | Low Risk | Low Risk | Some concerns | Some concerns |
| Grilo,2022 | Low Risk | Low Risk | Low Risk | Low Risk | Low Risk | Low Risk | Some concerns | Some concerns |
| Grilo,2023_a | Low Risk | Low Risk | Low Risk | Low Risk | Low Risk | Some concerns | Some concerns | Some concerns |
| Grilo,2023_b | Low Risk | Low Risk | Low Risk | Low Risk | Low Risk | Some concerns | Some concerns | Some concerns |
| McElroy,2003 | Some concerns | Some concerns | Low Risk | Some concerns | Some concerns | Some concerns | Some concerns | Some concerns |
| McElroy,2007 | Low Risk | Some concerns | Low Risk | Some concerns | Low Risk | Low Risk | Some concerns | Some concerns |
| Claudino,2007 | Low Risk | Low Risk | Low Risk | Low Risk | Low Risk | Low Risk | Some concerns | Some concerns |

**Supplement S5. Publication bias (funnel plot)**


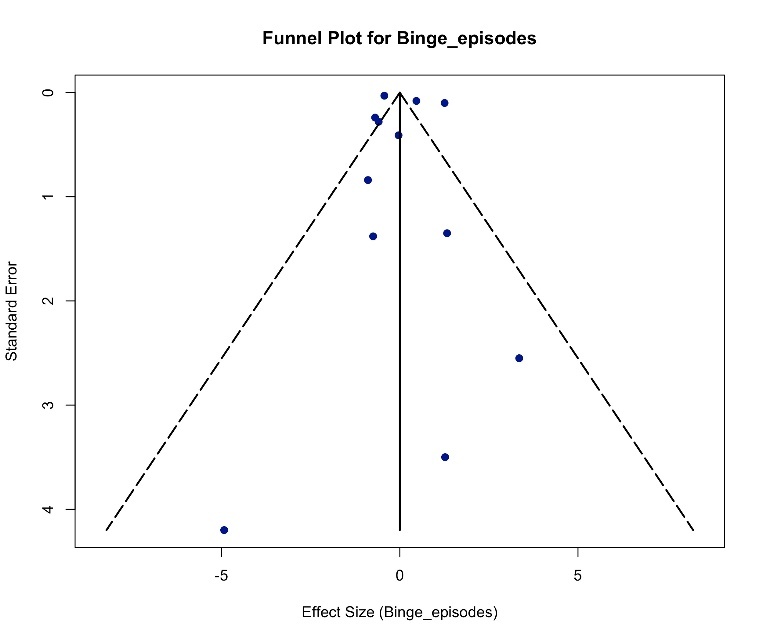
Figure S5.1: binge episodes (frequency)


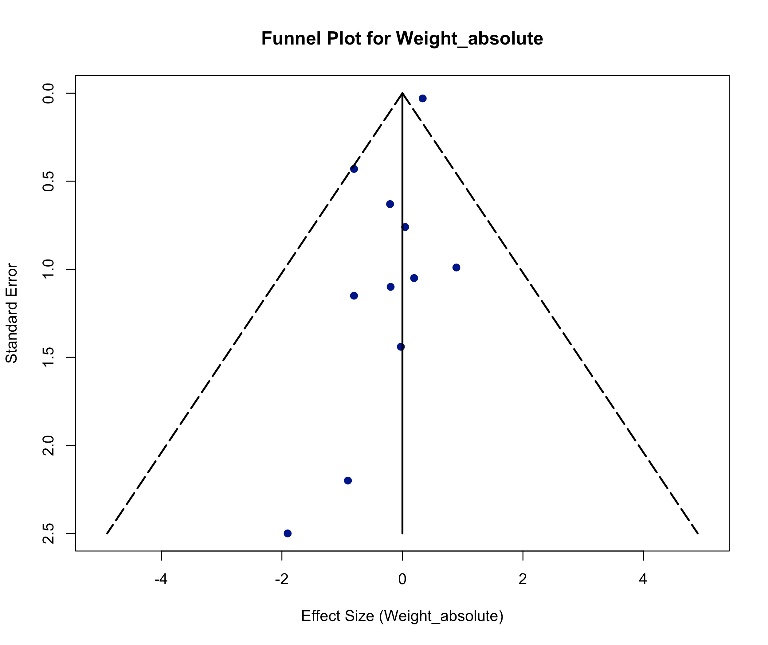
Figure S5.2: Change in weight


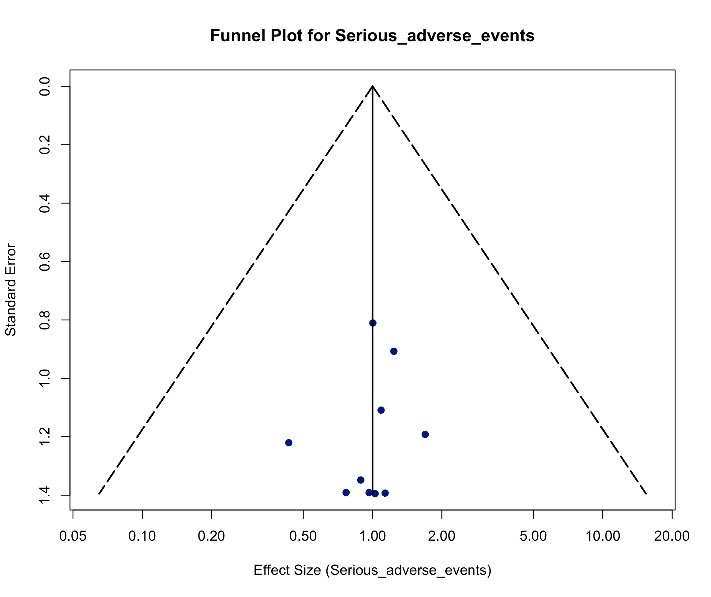
Figure S5.3: Serious adverse events

Figure S5.4: Treatment discontinuation


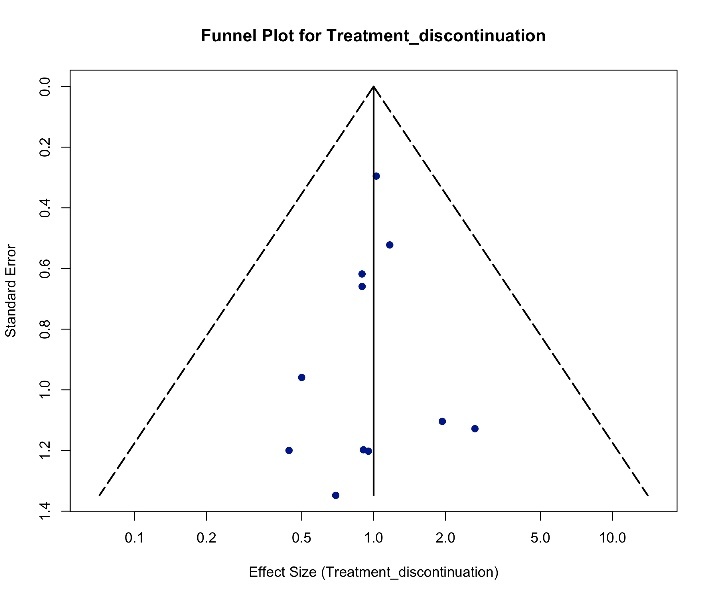


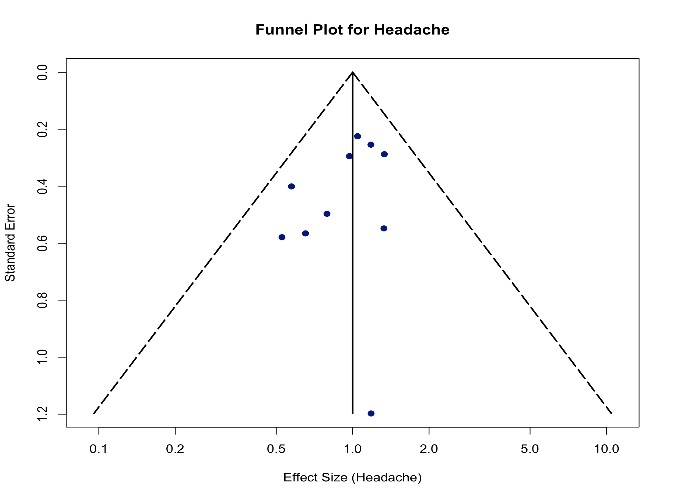
Figure S5.5: Headache

Figure S5.6: Dry mouth


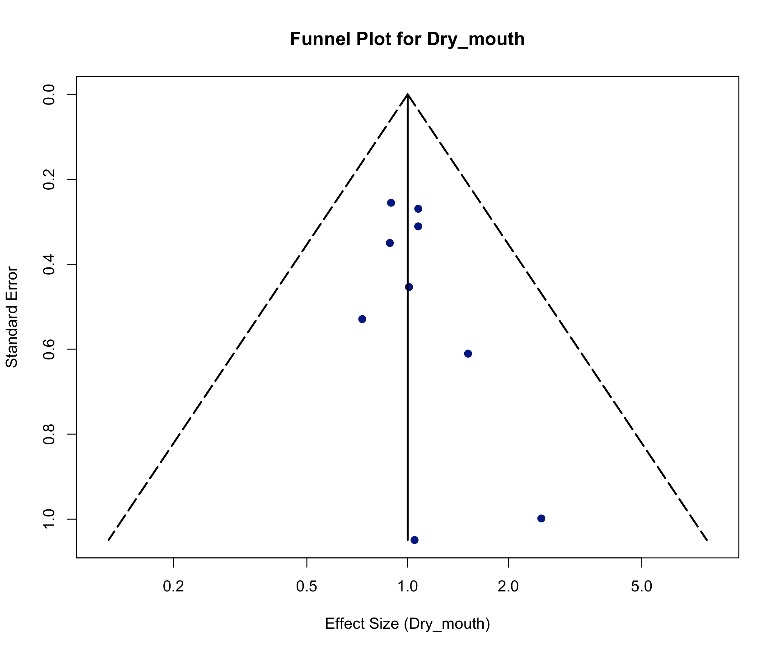


Figure S5.7: Gastrointestinal adverse events


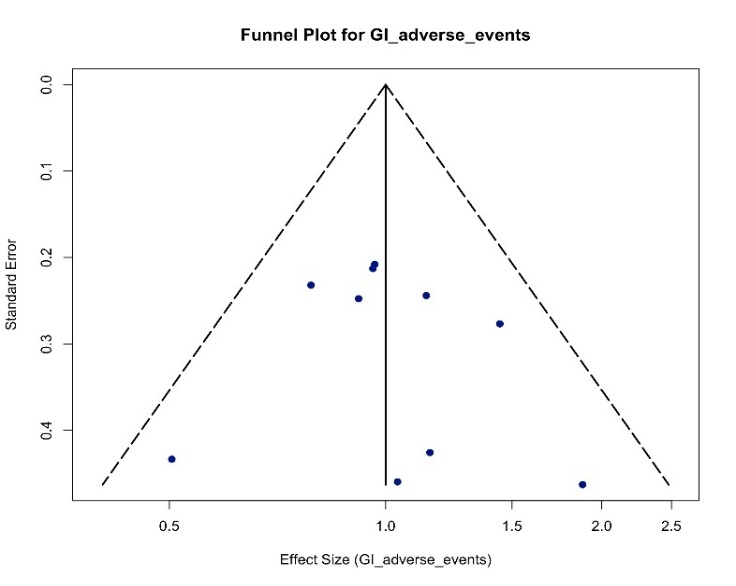


**Supplement S6: League table for each outcome**

Table S6.1: League table for binge episodes (frequency)

|  | Naltrexone / bupropion | Topiramate | lisdexamfetamine | Placebo |
| --- | --- | --- | --- | --- |
| Naltrexone / bupropion | Naltrexone / bupropion |  |  | -2.14 (-4.30; 0.03) |
| Topiramate | -0.82 (-2.99; 1.34) | Topiramate |  | -1.31 (-1.37; -1.26) |
| lisdexamfetamine | -1.33 (-3.50; 0.85) | -0.50 (-0.68; -0.33) | lisdexamfetamine | -0.81 (-0.98; -0.64) |
| Placebo | -2.14 (-4.30; 0.03) | -1.31 (-1.37; -1.26) | -0.81 (-0.98; -0.64) | Placebo |

Table S6.2: League table for weight change

|  | Topiramate | lisdexamfetamine | Naltrexone / bupropion | Placebo |
| --- | --- | --- | --- | --- |
| Topiramate | Topiramate | . | . | -5.83 (-5.89; -5.78) |
| lisdexamfetamine | -1.24 (-2.06; -0.41) | lisdexamfetamine | . | -4.60 (-5.42; -3.77) |
| Naltrexone / bupropion | -2.26 (-3.58; -0.94) | -1.02 (-2.58; 0.53) | Naltrexone / bupropion | -3.57 (-4.89; -2.26) |
| Placebo | -5.83 (-5.89; -5.78) | -4.60 (-5.42; -3.77) | -3.57 (-4.89; -2.26) | Placebo |

Table S6.3: League table for serious adverse events

|  | Naltrexone / bupropion | Placebo | Topiramate | lisdexamfetamine |
| --- | --- | --- | --- | --- |
| Naltrexone / bupropion | Naltrexone / bupropion | 0.94 (0.14; 6.26) | . | . |
| Placebo | 0.94 (0.14; 6.26) | Placebo | 1.00 (0.29; 3.42) | 0.85 (0.31; 2.28) |
| Topiramate | 0.94 (0.10; 9.00) | 1.00 (0.29; 3.42) | Topiramate | . |
| lisdexamfetamine | 0.79 (0.09; 6.75) | 0.85 (0.31; 2.28) | 0.85 (0.17; 4.09) | lisdexamfetamine |

Table S6.4: League table for Treatment discontinuation

|  | Placebo | Naltrexone / bupropion | Topiramate | lisdexamfetamine |
| --- | --- | --- | --- | --- |
| Placebo | Placebo | 0.83 (0.21; 3.30) | 0.54 (0.32; 0.91) | 0.50 (0.26; 0.96) |
| Naltrexone / bupropion | 0.83 (0.21; 3.30) | Naltrexone / bupropion | . | . |
| Topiramate | 0.54 (0.32; 0.91) | 0.65 (0.15; 2.82) | Topiramate | . |
| lisdexamfetamine | 0.50 (0.26; 0.96) | 0.60 (0.13; 2.76) | 0.92 (0.40; 2.13) | lisdexamfetamine |

Table S6.5: League table for Headache

|  | Placebo | Topiramate | lisdexamfetamine | Naltrexone / bupropion |
| --- | --- | --- | --- | --- |
| Placebo | Placebo | 0.98 (0.73; 1.33) | 0.65 (0.46; 0.93) | 0.56 (0.27; 1.14) |
| Topiramate | 0.98 (0.73; 1.33) | Topiramate | . | . |
| lisdexamfetamine | 0.65 (0.46; 0.93) | 0.66 (0.42; 1.06) | lisdexamfetamine | . |
| Naltrexone / bupropion | 0.56 (0.27; 1.14) | 0.57 (0.26; 1.24) | 0.85 (0.38; 1.90) | Naltrexone / bupropion |

Table S6.6: League table for Dry mouth

|  | Placebo | Topiramate | Naltrexone / bupropion | lisdexamfetamine |
| --- | --- | --- | --- | --- |
| Placebo | Placebo | 0.76 (0.50; 1.15) | 0.43 (0.20; 0.94) | 0.19 (0.14; 0.27) |
| Topiramate | 0.76 (0.50; 1.15) | Topiramate | . | . |
| Naltrexone / bupropion | 0.43 (0.20; 0.94) | 0.56 (0.23; 1.37) | Naltrexone / bupropion | . |
| lisdexamfetamine | 0.19 (0.14; 0.27) | 0.25 (0.15; 0.44) | 0.45 (0.19; 1.06) | lisdexamfetamine |

Table S6.7: League table for Gastrointestinal adverse events

|  | Placebo | Topiramate | Naltrexone / bupropion | lisdexamfetamine |
| --- | --- | --- | --- | --- |
| Placebo | Placebo | 0.85 (0.66; 1.11) | 0.63 (0.43; 0.92) | 0.44 (0.33; 0.59) |
| Topiramate | 0.85 (0.66; 1.11) | Topiramate | . | . |
| Naltrexone / bupropion | 0.63 (0.43; 0.92) | 0.74 (0.47; 1.17) | Naltrexone / bupropion | . |
| lisdexamfetamine | 0.44 (0.33; 0.59) | 0.52 (0.35; 0.77) | 0.70 (0.44; 1.12) | lisdexamfetamine |

**Supplement S7: Treatment ranking for each outcome**

Table S7.1: Treatment ranking for binge episodes (frequency)

| **Rank** | **Intervention** | **P-score** |
| --- | --- | --- |
| First | Naltrexone / bupropion | 0.7430 |
| Second | Topiramate | 0.6287 |
| Third | lisdexamfetamine | 0.6135 |
| Forth | Placebo | 0.0148 |

| **Rank** | **Intervention** | **P-score** |
| --- | --- | --- |
| First | Topiramate | 0.9842 |
| Second | lisdexamfetamine | 0.6441 |
| Third | Naltrexone / bupropion | 0.3717 |
| Forth | Placebo | 0.0000 |

Table S7.2: Treatment ranking for change in weight

Table S7.3: Treatment ranking for serious adverse events

| **Rank** | **Intervention** | **P-score** |
| --- | --- | --- |
| First | Naltrexone / bupropion | 0.5441 |
| Second | Placebo | 0.5337 |
| Third | Topiramate | 0.5207 |
| Forth | lisdexamfetamine | 0.4015 |

Table S7.4: Treatment ranking for Treatment discontinuation

| **Rank** | **Intervention** | **P-score** |
| --- | --- | --- |
| First | Placebo | 0.8579 |
| Second | Naltrexone / bupropion | 0.6198 |
| Third | Topiramate | 0.2881 |
| Forth | lisdexamfetamine | 0.2342 |

| **Rank** | **Intervention** | **P-score** |
| --- | --- | --- |
| First | Placebo | 0.8267 |
| Second | Topiramate | 0.7788 |
| Third | lisdexamfetamine | 0.2346 |
| Forth | Naltrexone / bupropion | 0.1599 |

Table S7.5: Treatment ranking for Headache

Table S7.6: Treatment ranking for Dry mouth

| **Rank** | **Intervention** | **P-score** |
| --- | --- | --- |
| First | Placebo | 0.9622 |
| Second | Topiramate | 0.6644 |
| Third | Naltrexone / bupropion | 0.3621 |
| Forth | lisdexamfetamine | 0.0113 |

Table S7.7: Treatment ranking for Gastrointestinal adverse events

| **Rank** | **Intervention** | **P-score** |
| --- | --- | --- |
| First | Placebo | 0.9509 |
| Second | Topiramate | 0.6733 |
| Third | Naltrexone / bupropion | 0.3471 |
| Forth | lisdexamfetamine | 0.0287 |

**Supplement S8: Certainty of evidence for each outcome**

Table S8.1 Binge episodes (frequency)

| Comparison | Within Study Bias | Reporting Bias | Indirectness | Imprecision | Heterogeneity | Incoherence | Overall Confidence | Reason(s) for downgrading |
| --- | --- | --- | --- | --- | --- | --- | --- | --- |
| Placebo:Topiramate | Some concerns | Some concerns | No concerns | No concerns | Major concerns | No concerns | Low | Within-study bias, Reporting bias, Heterogeneity |
| Naltrexone / bupropion:Placebo | Some concerns | Some concerns | Some concerns | Major concerns | Major concerns | No concerns | Very low | Within-study bias, Reporting bias, Indirectness, Imprecision, Heterogeneity |
| lisdexamfetamine:Placebo | Some concerns | Some concerns | Some concerns | No concerns | Major concerns | No concerns | Low | Within-study bias, Reporting bias, Indirectness, Heterogeneity |
| Naltrexone / bupropion:Topiramate | Some concerns | Some concerns | Some concerns | Major concerns | Major concerns | No concerns | Very low | Within-study bias, Reporting bias, Indirectness, Imprecision, Heterogeneity |
| lisdexamfetamine:Topiramate | Some concerns | Some concerns | Some concerns | Major concerns | Major concerns | No concerns | Very low | Within-study bias, Reporting bias, Indirectness, Imprecision, Heterogeneity |
| lisdexamfetamine:Naltrexone / bupropion | Some concerns | Some concerns | Some concerns | Major concerns | Major concerns | No concerns | Very low | Within-study bias, Reporting bias, Indirectness, Imprecision, Heterogeneity |

Table S8.2: Change in weight

| Comparison | Within Study Bias | Reporting Bias | Indirectness | Imprecision | Heterogeneity | Incoherence | Overall Confidence | Reason(s) for downgrading |
| --- | --- | --- | --- | --- | --- | --- | --- | --- |
| Placebo:Topiramate | Some concerns | Some concerns | No concerns | No concerns | Some concerns | No concerns | Low | Within-study bias, Reporting bias, Heterogeneity |
| Naltrexone / bupropion:Placebo | Some concerns | Some concerns | Some concerns | Some concerns | Some concerns | No concerns | Low | Within-study bias, Reporting bias, Indirectness, Imprecision, Heterogeneity |
| lisdexamfetamine:Placebo | Some concerns | Some concerns | Some concerns | No concerns | Some concerns | No concerns | Low | Within-study bias, Reporting bias, Indirectness, Heterogeneity |
| Naltrexone / bupropion:Topiramate | Some concerns | Some concerns | Some concerns | Some concerns | Some concerns | No concerns | Low | Within-study bias, Reporting bias, Indirectness, Imprecision, Heterogeneity |
| lisdexamfetamine:Topiramate | Some concerns | Some concerns | Some concerns | Major concerns | Some concerns | No concerns | Low | Within-study bias, Reporting bias, Indirectness, Imprecision, Heterogeneity |
| lisdexamfetamine:Naltrexone / bupropion | Some concerns | Some concerns | Some concerns | Major concerns | Some concerns | No concerns | Low | Within-study bias, Reporting bias, Indirectness, Imprecision, Heterogeneity |

Table S8.3: Serious adverse events

| Comparison | Within Study Bias | Reporting Bias | Indirectness | Imprecision | Heterogeneity | Incoherence | Overall Confidence | Reason(s) for downgrading |
| --- | --- | --- | --- | --- | --- | --- | --- | --- |
| Placebo:Topiramate | Some concerns | Some concerns | No concerns | Major concerns | No concerns | No concerns | Low | Within-study bias, Reporting bias, Imprecision |
| Naltrexone / bupropion:Placebo | Some concerns | Some concerns | Some concerns | Major concerns | No concerns | No concerns | Low | Within-study bias, Reporting bias, Indirectness, Imprecision |
| lisdexamfetamine:Placebo | Some concerns | Some concerns | Some concerns | No concerns | No concerns | No concerns | Low | Within-study bias, Reporting bias, Indirectness |
| Naltrexone / bupropion:Topiramate | Some concerns | Some concerns | Some concerns | Major concerns | No concerns | No concerns | Low | Within-study bias, Reporting bias, Indirectness, Imprecision |
| lisdexamfetamine:Topiramate | Some concerns | Some concerns | Some concerns | Major concerns | No concerns | No concerns | Low | Within-study bias, Reporting bias, Indirectness, Imprecision |
| lisdexamfetamine:Naltrexone / bupropion | Some concerns | Some concerns | Some concerns | Major concerns | No concerns | No concerns | Low | Within-study bias, Reporting bias, Indirectness, Imprecision |

Table S8.4: Treatment discontinuation

| Comparison | Within Study Bias | Reporting Bias | Indirectness | Imprecision | Heterogeneity | Incoherence | Overall Confidence | Reason(s) for downgrading |
| --- | --- | --- | --- | --- | --- | --- | --- | --- |
| Placebo:Topiramate | Some concerns | Some concerns | No concerns | Major concerns | No concerns | No concerns | Low | Within-study bias, Reporting bias, Imprecision |
| Naltrexone / bupropion:Placebo | Some concerns | Some concerns | Some concerns | Major concerns | No concerns | No concerns | Low | Within-study bias, Reporting bias, Indirectness, Imprecision |
| lisdexamfetamine:Placebo | Some concerns | Some concerns | Some concerns | No concerns | No concerns | No concerns | Low | Within-study bias, Reporting bias, Indirectness |
| Naltrexone / bupropion:Topiramate | Some concerns | Some concerns | Some concerns | Major concerns | No concerns | No concerns | Low | Within-study bias, Reporting bias, Indirectness, Imprecision |
| lisdexamfetamine:Topiramate | Some concerns | Some concerns | Some concerns | No concerns | No concerns | No concerns | Low | Within-study bias, Reporting bias, Indirectness |
| lisdexamfetamine:Naltrexone / bupropion | Some concerns | Some concerns | Some concerns | Major concerns | No concerns | No concerns | Low | Within-study bias, Reporting bias, Indirectness, Imprecision |

Table S8.5: Headache

| Comparison | Within Study Bias | Reporting Bias | Indirectness | Imprecision | Heterogeneity | Incoherence | Overall Confidence | Reason(s) for downgrading |
| --- | --- | --- | --- | --- | --- | --- | --- | --- |
| Placebo:Topiramate | Some concerns | Some concerns | No concerns | No concerns | No concerns | No concerns | Moderate | Within-study bias, Reporting bias |
| Naltrexone / bupropion:Placebo | Some concerns | Some concerns | Some concerns | No concerns | No concerns | No concerns | Low | Within-study bias, Reporting bias, Indirectness |
| lisdexamfetamine:Placebo | Some concerns | Some concerns | Some concerns | No concerns | No concerns | No concerns | Low | Within-study bias, Reporting bias, Indirectness |
| Naltrexone / bupropion:Topiramate | Some concerns | Some concerns | Some concerns | No concerns | No concerns | No concerns | Low | Within-study bias, Reporting bias, Indirectness |
| lisdexamfetamine:Topiramate | Some concerns | Some concerns | Some concerns | No concerns | No concerns | No concerns | Low | Within-study bias, Reporting bias, Indirectness |
| lisdexamfetamine:Naltrexone / bupropion | Some concerns | Some concerns | Some concerns | No concerns | No concerns | No concerns | Low | Within-study bias, Reporting bias, Indirectness |

Table S8.6: Dry mouth

| Comparison | Within Study Bias | Reporting Bias | Indirectness | Imprecision | Heterogeneity | Incoherence | Overall Confidence | Reason(s) for downgrading |
| --- | --- | --- | --- | --- | --- | --- | --- | --- |
| Naltrexone / bupropion:Placebo | Some concerns | Some concerns | Some concerns | No concerns | No concerns | No concerns | Low | Within-study bias, Reporting bias, Indirectness |
| lisdexamfetamine:Placebo | Some concerns | Some concerns | Some concerns | No concerns | No concerns | No concerns | Low | Within-study bias, Reporting bias, Indirectness |
| Placebo:Topiramate | Some concerns | Some concerns | No concerns | No concerns | No concerns | No concerns | Moderate | Within-study bias, Reporting bias |
| lisdexamfetamine:Naltrexone / bupropion | Some concerns | Some concerns | Some concerns | No concerns | No concerns | No concerns | Low | Within-study bias, Reporting bias, Indirectness |
| Naltrexone / bupropion:Topiramate | Some concerns | Some concerns | No concerns | No concerns | No concerns | No concerns | Moderate | Within-study bias, Reporting bias |
| lisdexamfetamine:Topiramate | Some concerns | Some concerns | Some concerns | No concerns | No concerns | No concerns | Low | Within-study bias, Reporting bias, Indirectness |

Table S8.7: Gastrointestinal adverse events

| Comparison | Within Study Bias | Reporting Bias | Indirectness | Imprecision | Heterogeneity | Incoherence | Overall Confidence | Reason(s) for downgrading |
| --- | --- | --- | --- | --- | --- | --- | --- | --- |
| Placebo:Topiramate | Some concerns | Some concerns | No concerns | No concerns | No concerns | No concerns | Moderate | Within-study bias, Reporting bias |
| Naltrexone / bupropion:Placebo | Some concerns | Some concerns | Some concerns | No concerns | No concerns | No concerns | Low | Within-study bias, Reporting bias, Indirectness |
| lisdexamfetamine:Placebo | Some concerns | Some concerns | Some concerns | No concerns | No concerns | No concerns | Low | Within-study bias, Reporting bias, Indirectness |
| Naltrexone / bupropion:Topiramate | Some concerns | Some concerns | Some concerns | No concerns | No concerns | No concerns | Low | Within-study bias, Reporting bias, Indirectness |
| lisdexamfetamine:Topiramate | Some concerns | Some concerns | Some concerns | No concerns | No concerns | No concerns | Low | Within-study bias, Reporting bias, Indirectness |
| lisdexamfetamine:Naltrexone / bupropion | Some concerns | Some concerns | Some concerns | No concerns | No concerns | No concerns | Low | Within-study bias, Reporting bias, Indirectness |

**Supplement S9:** Sensitivity analysis

Figure S9.1.1 binge episodes (frequency) for large sample studies. Network geometry

**
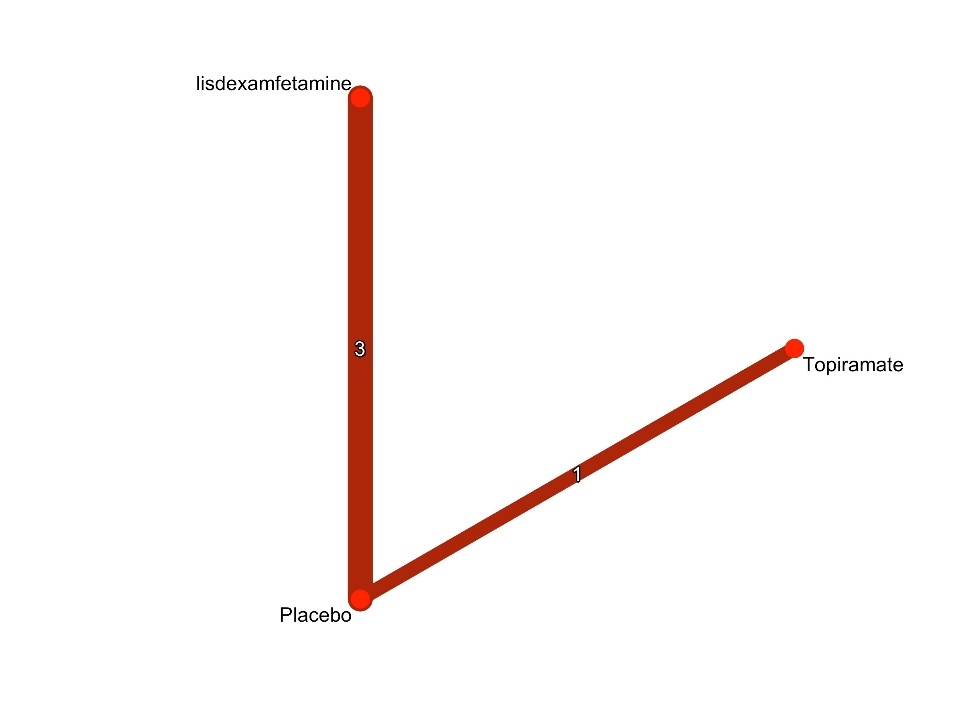
**

Figure S9.1.2 binge episodes (frequency) for large sample studies. Funnel plot

**
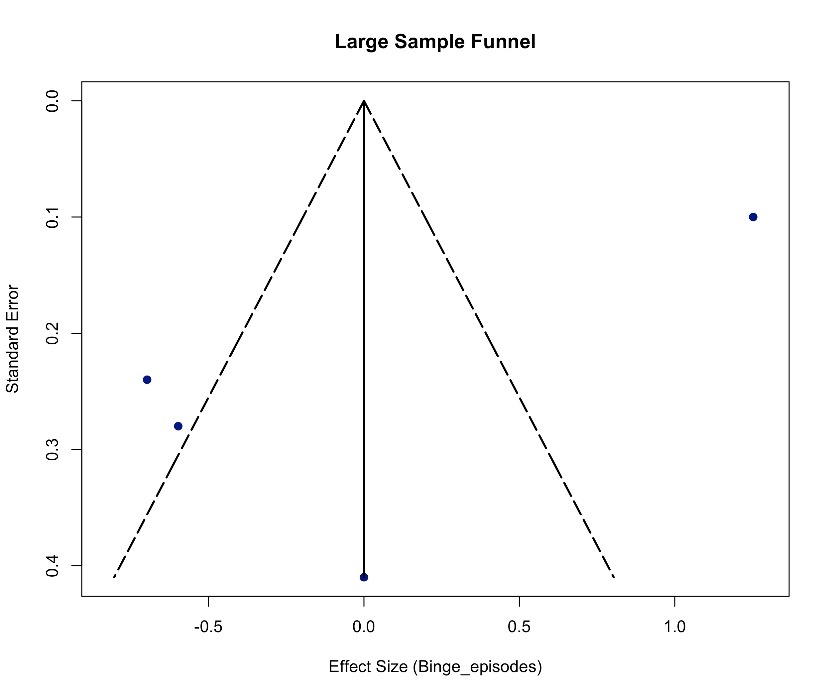
**

Figure S9.1.3 binge episodes (frequency) for large sample studies. Forest plot


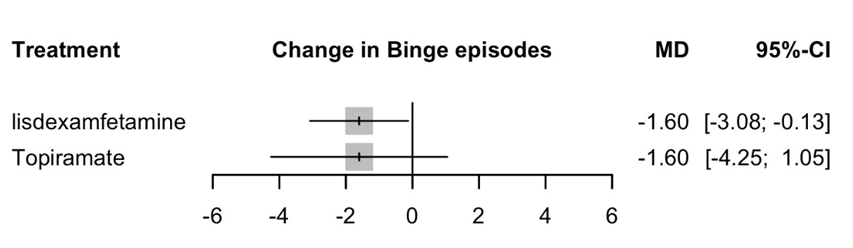


Figure S9.2.1 binge episodes (frequency) for low-risk studies. Network geometry

**
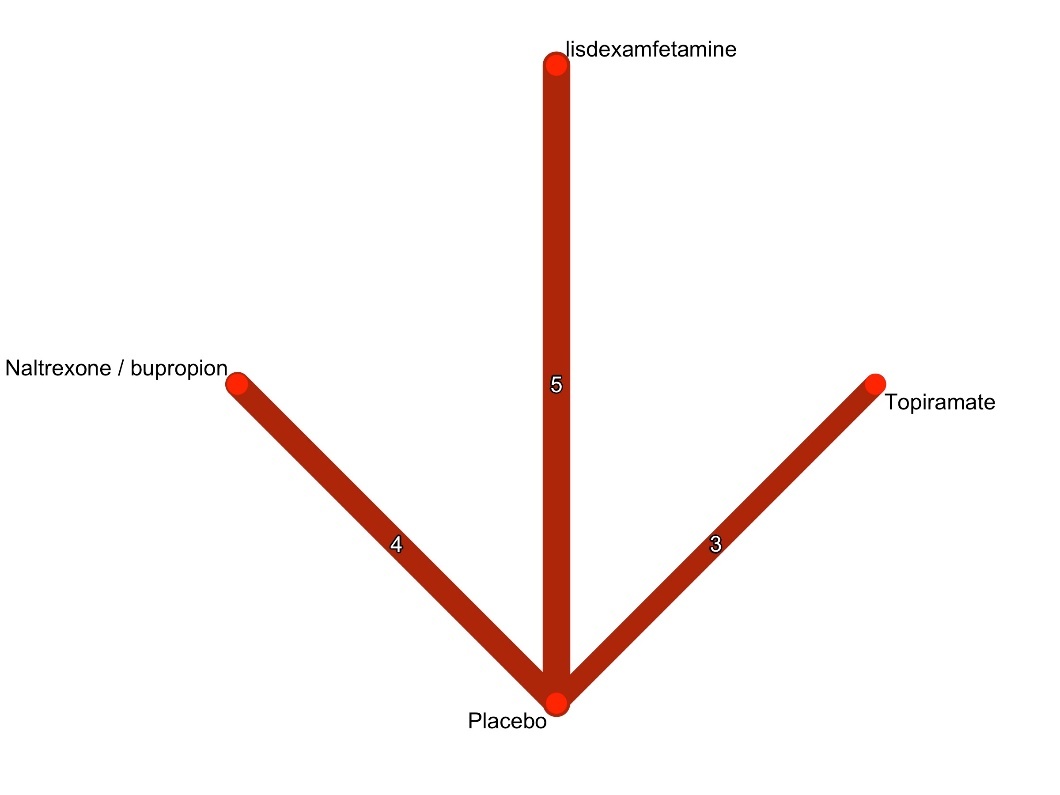
**

Figure S9.2.2 binge episodes (frequency) for low-risk studies. Funnel plot

**
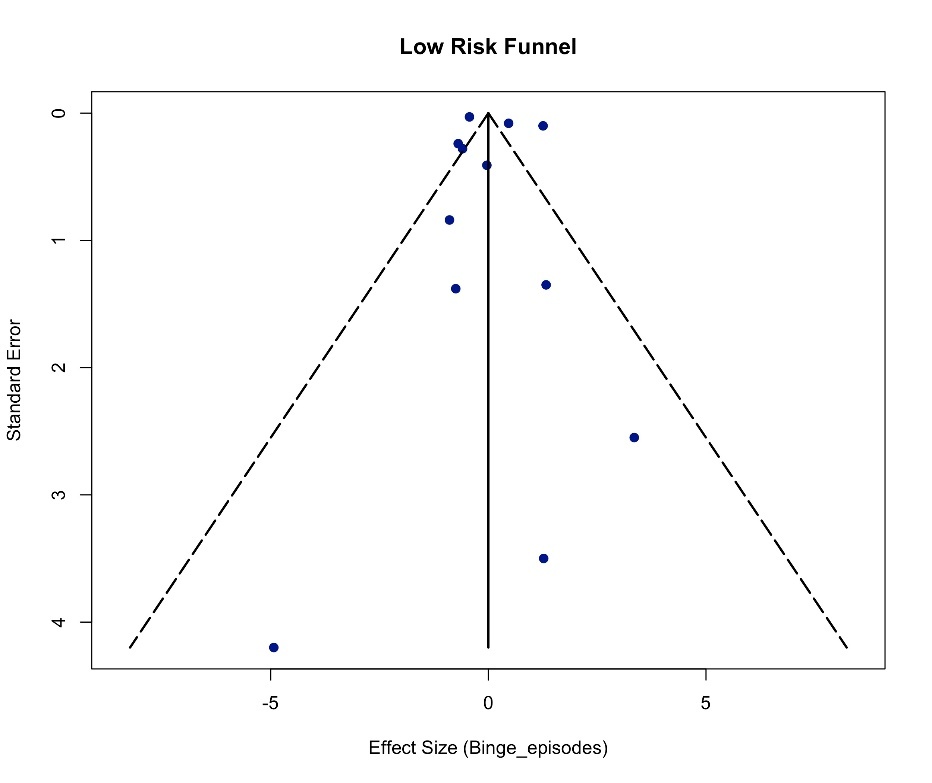
**

Figure S9.2.3 binge episodes (frequency) for large sample studies. Forest plot

**
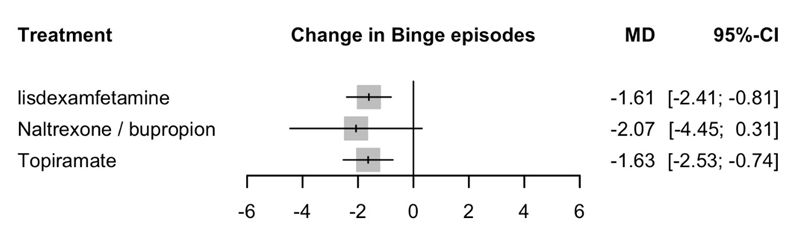
**

Figure S9.3.1 weight for large sample studies. Network geometry

**
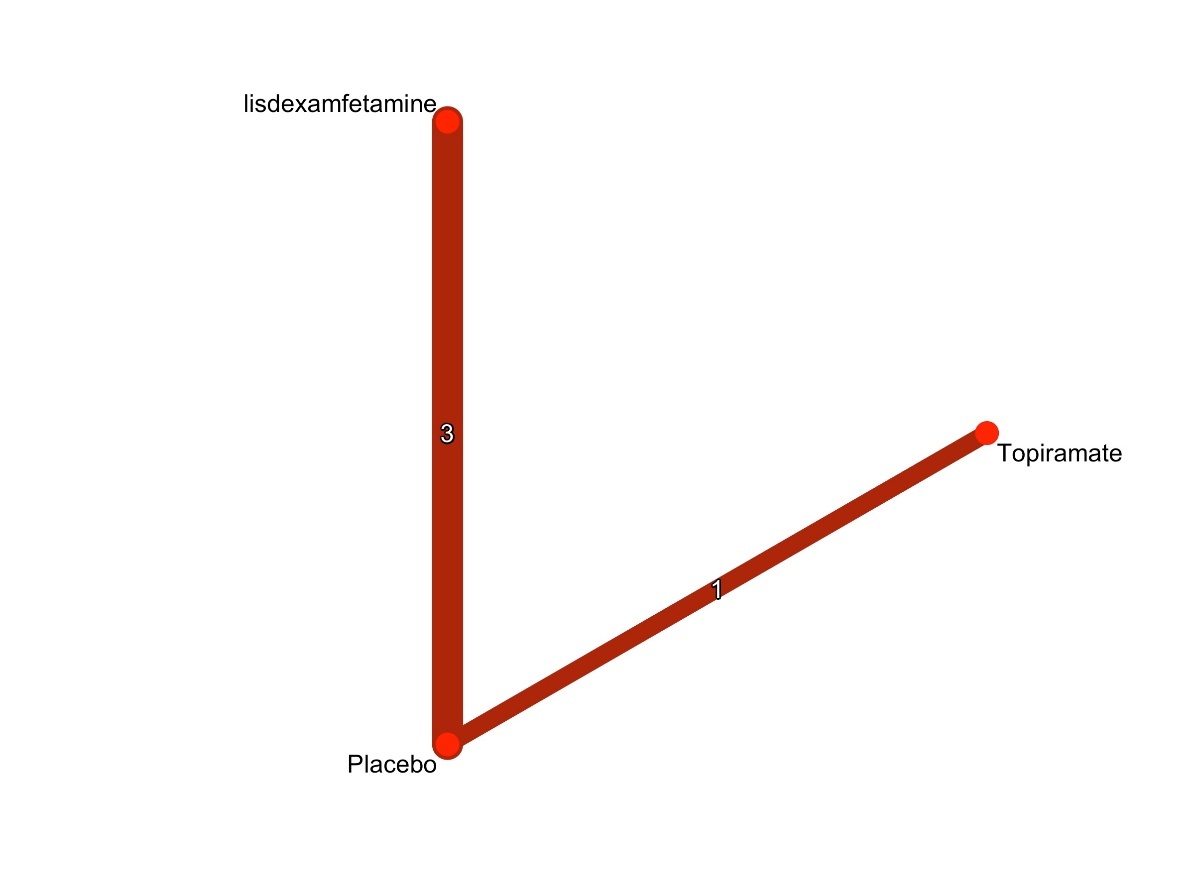
**

Figure S9.3.2 weight for large sample studies. Funnel plot

**
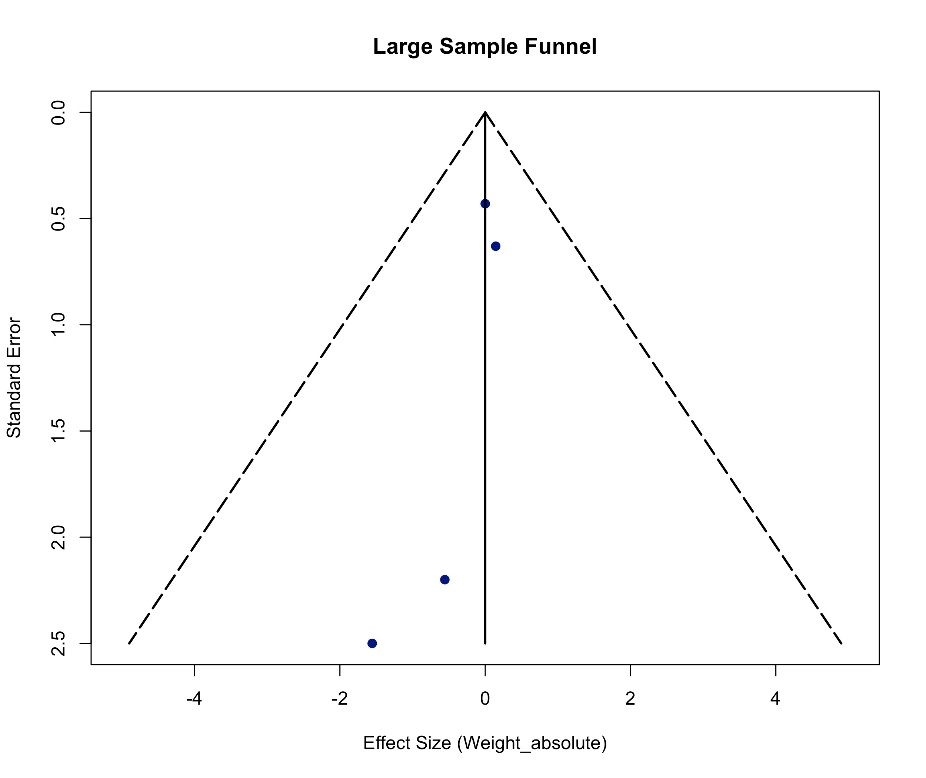
**

Figure S9.3.3 weight for large sample studies. Forest plot

**
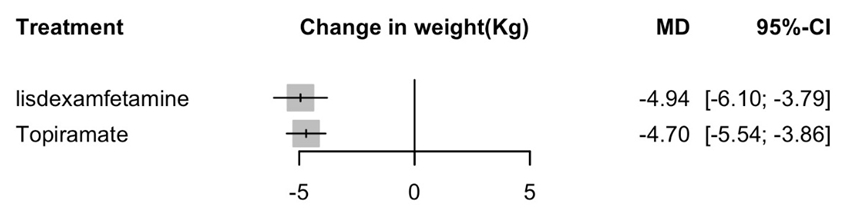
**

Figure S9.4.1 weight for low-risk studies. Network geometry

**
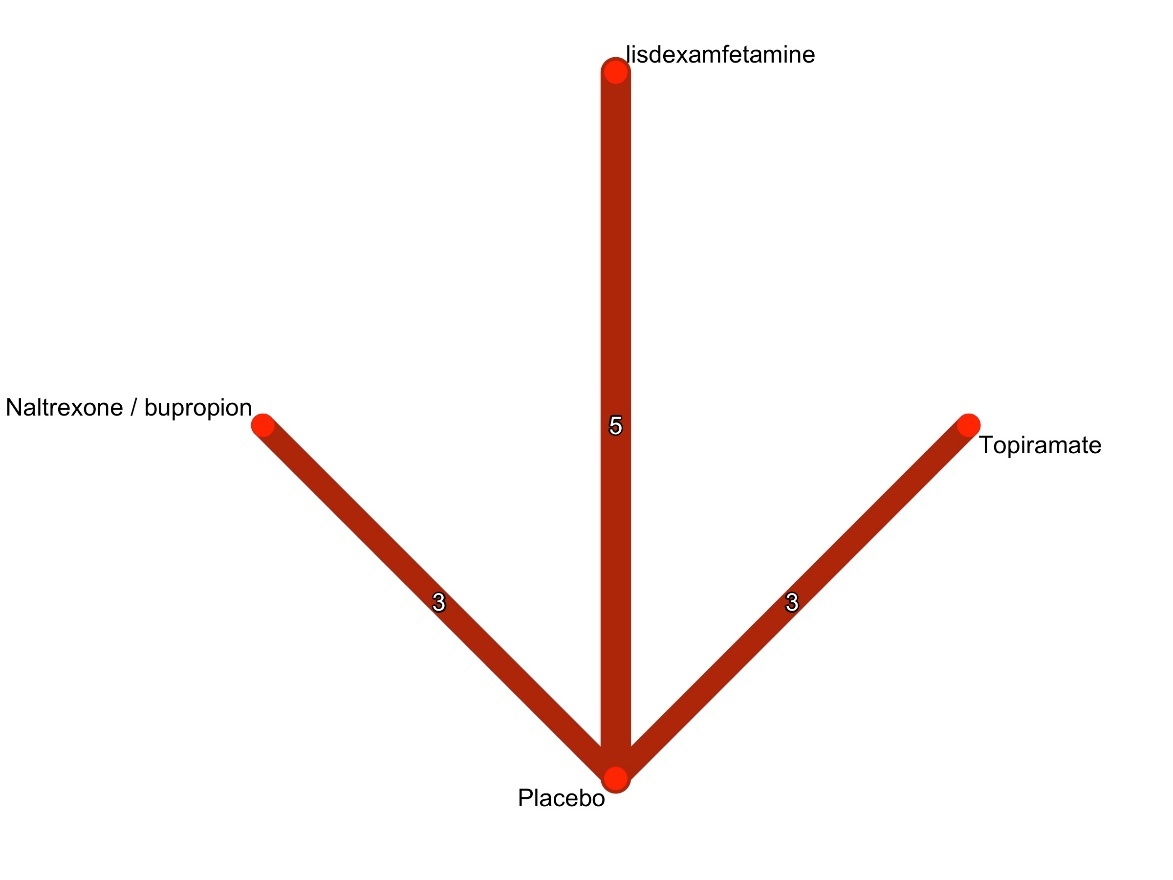
**

**
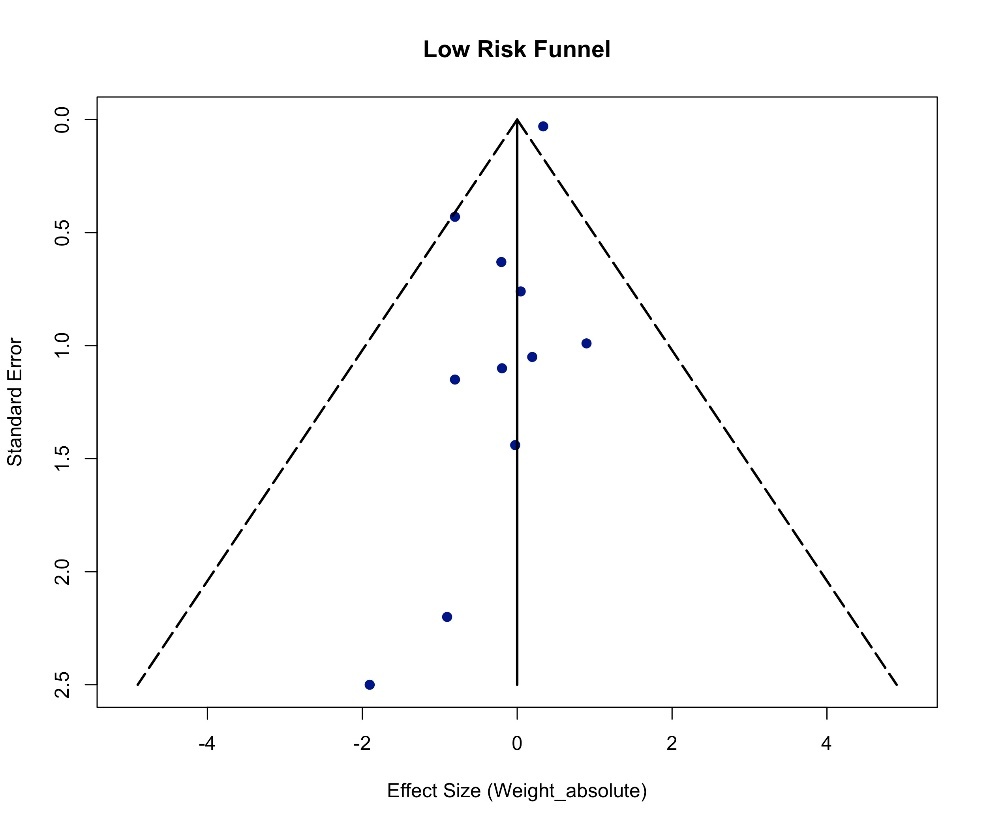
**Figure S9.4.2 weight for low-risk studies. Funnel plot

Figure S9.4.3 weight for low-risk studies. Forest plot

**
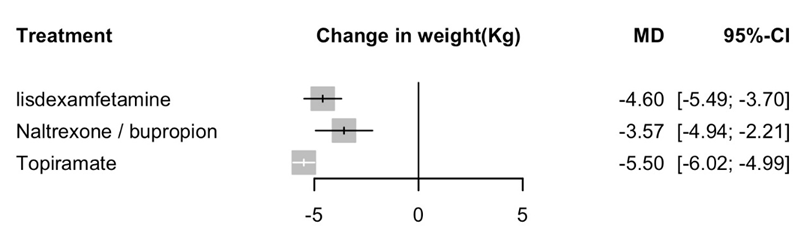
**

| Outcome | Study Omitted | I^2 | tau^2 | Interpretation |
| --- | --- | --- | --- | --- |
| Bing episodes | Claudino,2007 | 91.4% | 1.3208 | Effect stable |
| Bing episodes | Grilo,2021 | 96% | 0.5747 | Effect stable |
| Bing episodes | Grilo,2022 | 96.1% | 0.5785 | Effect stable |
| Bing episodes | Grilo,2023_a | 96% | 0.5726 | Effect stable |
| Bing episodes | Grilo,2023_b | 96.1% | 0.5772 | Effect stable |
| Bing episodes | Grilo,2024 | 96.1% | 0.5799 | Effect stable |
| Bing episodes | Guerdjikova,2016 | 96% | 0.5705 | Effect stable |
| Bing episodes | McElroy,2003 | 91.4% | 1.3058 | Effect stable |
| Bing episodes | McElroy,2007 | 96.1% | 0.5960 | Effect stable |
| Bing episodes | McElroy,2015 | 93.2% | 0.3612 | Effect stable |
| Bing episodes | McElroy,2016_a1 | 95% | 0.4855 | Effect stable |
| Bing episodes | McElroy,2016_a2 | 95.5% | 0.5289 | Effect stable |

Table S9.5.1 leave one out analysis for binge episodes (frequency)

Table S9.5.2 Leave one out analysis for weight

| Outcome | Study Omitted | I^2 | tau^2 | Interpretation |
| --- | --- | --- | --- | --- |
| Weight | Claudino,2007 | 0% | 0 | Effect stable |
| Weight | Grilo,2022 | 27.9% | 0.1602 | Effect stable |
| Weight | Grilo,2023_a | 27.5% | 0.1592 | Effect stable |
| Weight | Grilo,2023_b | 27.5% | 0.1594 | Effect stable |
| Weight | Grilo,2024 | 27.9% | 0.1723 | Effect stable |
| Weight | Guerdjikova,2016 | 19.6% | 0.1048 | Effect stable |
| Weight | McElroy,2003 | 19.9% | 0.1087 | Effect stable |
| Weight | McElroy,2007 | 0% | 0 | Effect stable |
| Weight | McElroy,2015 | 26.5% | 0.1628 | Effect stable |
| Weight | McElroy,2016_a1 | 26.6% | 0.1473 | Effect stable |
| Weight | McElroy,2016_a2 | 23.2% | 0.1224 | Effect stable |

Table S9.5.3 Leave one out analysis for Serious adverse events

| Outcome | Study Omitted | I^2 | tau^2 | Interpretation |
| --- | --- | --- | --- | --- |
| Serious adverse events | Claudino,2007 | 0% | 0 | Effect stable |
| Serious adverse events | Grilo,2021 | 0% | 0 | Effect stable |
| Serious adverse events | Grilo,2023_b | 0% | 0 | Effect stable |
| Serious adverse events | Grilo,2024 | 0% | 0 | Effect stable |
| Serious adverse events | Guerdjikova,2016 | 0% | 0 | Effect stable |
| Serious adverse events | McElroy,2003 | 0% | 0 | Effect stable |
| Serious adverse events | McElroy,2007 | 0% | 0 | Effect stable |
| Serious adverse events | McElroy,2015 | 0% | 0 | Effect stable |
| Serious adverse events | McElroy,2016_a1 | 0% | 0 | Effect stable |
| Serious adverse events | McElroy,2016_a2 | 0% | 0 | Effect stable |

Table S9.5.4 Leave one out analysis for Treatment discontinuation

| Outcome | Study Omitted | I^2 | tau^2 | Interpretation |
| --- | --- | --- | --- | --- |
| Treatment discontinuation | Claudino,2007 | 0% | 0 | Effect stable |
| Treatment discontinuation | Grilo,2021 | 0% | 0 | Effect stable |
| Treatment discontinuation | Grilo,2022 | 0% | 0 | Effect stable |
| Treatment discontinuation | Grilo,2023_b | 0% | 0 | Effect stable |
| Treatment discontinuation | Grilo,2024 | 0% | 0 | Effect stable |
| Treatment discontinuation | Guerdjikova,2016 | 0% | 0 | Effect stable |
| Treatment discontinuation | McElroy,2003 | 0% | 0 | Effect stable |
| Treatment discontinuation | McElroy,2007 | 0% | 0 | Effect stable |
| Treatment discontinuation | McElroy,2015 | 0% | 0 | Effect stable |
| Treatment discontinuation | McElroy,2016_a1 | 0% | 0 | Effect stable |
| Treatment discontinuation | McElroy,2016_a2 | 0% | 0 | Effect stable |

Table S9.5.5 Leave one out analysis for Headache

| Outcome | Study Omitted | I^2 | tau^2 | Interpretation |
| --- | --- | --- | --- | --- |
| Headache | Claudino,2007 | 0% | 0 | Effect stable |
| Headache | Grilo,2021 | 0% | 0 | Effect stable |
| Headache | Grilo,2023_b | 0% | 0 | Effect stable |
| Headache | Grilo,2024 | 0% | 0 | Effect stable |
| Headache | Guerdjikova,2016 | 0% | 0 | Effect stable |
| Headache | McElroy,2003 | 0% | 0 | Effect stable |
| Headache | McElroy,2007 | 0% | 0 | Effect stable |
| Headache | McElroy,2015 | 0% | 0 | Effect stable |
| Headache | McElroy,2016_a1 | 0% | 0 | Effect stable |
| Headache | McElroy,2016_a2 | 0% | 0 | Effect stable |

Table S9.5.6 Leave one out analysis for Dry mouth

| Outcome | Study Omitted | I^2 | tau^2 | Interpretation |
| --- | --- | --- | --- | --- |
| Dry mouth | Grilo,2021 | 0% | 0 | Effect stable |
| Dry mouth | Grilo,2023_b | 0% | 0 | Effect stable |
| Dry mouth | Grilo,2024 | 0% | 0 | Effect stable |
| Dry mouth | Guerdjikova,2016 | 0% | 0 | Effect stable |
| Dry mouth | McElroy,2003 | 0% | 0 | Effect stable |
| Dry mouth | McElroy,2007 | 0% | 0 | Effect stable |
| Dry mouth | McElroy,2015 | 0% | 0 | Effect stable |
| Dry mouth | McElroy,2016_a1 | 0% | 0 | Effect stable |
| Dry mouth | McElroy,2016_a2 | 0% | 0 | Effect stable |

Table S9.5.7 Leave one out analysis for Gastrointestinal adverse events

| Outcome | Study Omitted | I^2 | tau^2 | Interpretation |
| --- | --- | --- | --- | --- |
| Gastrointestinal adverse events | Claudino,2007 | 18.4% | 0.0220 | Effect stable |
| Gastrointestinal adverse events | Grilo,2021 | 21.2% | 0.0229 | Effect stable |
| Gastrointestinal adverse events | Grilo,2023_b | 21.2% | 0.0229 | Effect stable |
| Gastrointestinal adverse events | Grilo,2024 | 22.6% | 0.0238 | Effect stable |
| Gastrointestinal adverse events | Guerdjikova,2016 | 0% | 0 | Effect stable |
| Gastrointestinal adverse events | McElroy,2003 | 22.3% | 0.0291 | Effect stable |
| Gastrointestinal adverse events | McElroy,2007 | 21% | 0.0257 | Effect stable |
| Gastrointestinal adverse events | McElroy,2015 | 0% | 0 | Effect stable |
| Gastrointestinal adverse events | McElroy,2016_a1 | 0.8% | 0.0008 | Effect stable |
| Gastrointestinal adverse events | McElroy,2016_a2 | 0% | 0 | Effect stable |

**Supplement S10: PRISMA check list**

| **Section and Topic** | **Item #** | **Checklist item** | **Location where item is reported** |
| --- | --- | --- | --- |
| **TITLE** | | |  |
| Title | 1 | Identify the report as a systematic review. | 1 |
| **ABSTRACT** | | |  |
| Abstract | 2 | See the PRISMA 2020 for Abstracts checklist. | 1 |
| **INTRODUCTION** | | |  |
| Rationale | 3 | Describe the rationale for the review in the context of existing knowledge. | 2 |
| Objectives | 4 | Provide an explicit statement of the objective(s) or question(s) the review addresses. | 2 |
| **METHODS** | | |  |
| Eligibility criteria | 5 | Specify the inclusion and exclusion criteria for the review and how studies were grouped for the syntheses. | 3 |
| Information sources | 6 | Specify all databases, registers, websites, organisations, reference lists and other sources searched or consulted to identify studies. Specify the date when each source was last searched or consulted. | 3 |
| Search strategy | 7 | Present the full search strategies for all databases, registers and websites, including any filters and limits used. | 3 |
| Selection process | 8 | Specify the methods used to decide whether a study met the inclusion criteria of the review, including how many reviewers screened each record and each report retrieved, whether they worked independently, and if applicable, details of automation tools used in the process. | 3,4 |
| Data collection process | 9 | Specify the methods used to collect data from reports, including how many reviewers collected data from each report, whether they worked independently, any processes for obtaining or confirming data from study investigators, and if applicable, details of automation tools used in the process. | 3,4 |
| Data items | 10a | List and define all outcomes for which data were sought. Specify whether all results that were compatible with each outcome domain in each study were sought (e.g. for all measures, time points, analyses), and if not, the methods used to decide which results to collect. | 4 |
|  | 10b | List and define all other variables for which data were sought (e.g. participant and intervention characteristics, funding sources). Describe any assumptions made about any missing or unclear information. | 4 |
| Study risk of bias assessment | 11 | Specify the methods used to assess risk of bias in the included studies, including details of the tool(s) used, how many reviewers assessed each study and whether they worked independently, and if applicable, details of automation tools used in the process. | 4 |
| Effect measures | 12 | Specify for each outcome the effect measure(s) (e.g. risk ratio, mean difference) used in the synthesis or presentation of results. | 4 |
| Synthesis methods | 13a | Describe the processes used to decide which studies were eligible for each synthesis (e.g. tabulating the study intervention characteristics and comparing against the planned groups for each synthesis (item #5)). | 4 |
|  | 13b | Describe any methods required to prepare the data for presentation or synthesis, such as handling of missing summary statistics, or data conversions. | 4 |
|  | 13c | Describe any methods used to tabulate or visually display results of individual studies and syntheses. | 4 |
|  | 13d | Describe any methods used to synthesize results and provide a rationale for the choice(s). If meta-analysis was performed, describe the model(s), method(s) to identify the presence and extent of statistical heterogeneity, and software package(s) used. | 4 |
|  | 13e | Describe any methods used to explore possible causes of heterogeneity among study results (e.g. subgroup analysis, meta-regression). | 4 |
|  | 13f | Describe any sensitivity analyses conducted to assess robustness of the synthesized results. | 4 |
| Reporting bias assessment | 14 | Describe any methods used to assess risk of bias due to missing results in a synthesis (arising from reporting biases). | 4 |
| Certainty assessment | 15 | Describe any methods used to assess certainty (or confidence) in the body of evidence for an outcome. | 4 |
| **RESULTS** | | |  |
| Study selection | 16a | Describe the results of the search and selection process, from the number of records identified in the search to the number of studies included in the review, ideally using a flow diagram. | 5, supplement, figure 1 |
|  | 16b | Cite studies that might appear to meet the inclusion criteria, but which were excluded, and explain why they were excluded. | 5 |
| Study characteristics | 17 | Cite each included study and present its characteristics. | 5, supplement |
| Risk of bias in studies | 18 | Present assessments of risk of bias for each included study. | 5, supplement |
| Results of individual studies | 19 | For all outcomes, present, for each study: (a) summary statistics for each group (where appropriate) and (b) an effect estimate and its precision (e.g. confidence/credible interval), ideally using structured tables or plots. | 12-16 |
| Results of syntheses | 20a | For each synthesis, briefly summarise the characteristics and risk of bias among contributing studies. | 12-16 |
|  | 20b | Present results of all statistical syntheses conducted. If meta-analysis was done, present for each the summary estimate and its precision (e.g. confidence/credible interval) and measures of statistical heterogeneity. If comparing groups, describe the direction of the effect. | 12-16 |
|  | 20c | Present results of all investigations of possible causes of heterogeneity among study results. | 12-16 |
|  | 20d | Present results of all sensitivity analyses conducted to assess the robustness of the synthesized results. | 12-16, supplement |
| Reporting biases | 21 | Present assessments of risk of bias due to missing results (arising from reporting biases) for each synthesis assessed. | 5, supplement |
| Certainty of evidence | 22 | Present assessments of certainty (or confidence) in the body of evidence for each outcome assessed. | 5, supplement |
| **DISCUSSION** | | |  |
| Discussion | 23a | Provide a general interpretation of the results in the context of other evidence. |  |
|  | 23b | Discuss any limitations of the evidence included in the review. | 17-19 |
|  | 23c | Discuss any limitations of the review processes used. | 17-19 |
|  | 23d | Discuss implications of the results for practice, policy, and future research. | 17-19 |
| **OTHER INFORMATION** | | |  |
| Registration and protocol | 24a | Provide registration information for the review, including register name and registration number, or state that the review was not registered. | 3 |
|  | 24b | Indicate where the review protocol can be accessed, or state that a protocol was not prepared. | 3 |
|  | 24c | Describe and explain any amendments to information provided at registration or in the protocol. | 3 |
| Support | 25 | Describe sources of financial or non-financial support for the review, and the role of the funders or sponsors in the review. | 20 |
| Competing interests | 26 | Declare any competing interests of review authors. | 20 |
| Availability of data, code and other materials | 27 | Report which of the following are publicly available and where they can be found: template data collection forms; data extracted from included studies; data used for all analyses; analytic code; any other materials used in the review. | 20 |
